# Framework-Controlled Axial Coordination of Guest Molecules in Metalloporphyrin-Based MOFs

**DOI:** 10.1021/acs.inorgchem.6c00778

**Published:** 2026-07-16

**Authors:** Alison Arissa, Nicole Lahanas, Maaz Afzal, Roger Lalancette, Pavel Kucheryavy, Jenny V. Lockard

**Affiliations:** Department of Chemistry, 67206 Rutgers University, Newark, New Jersey 07102, United States

## Abstract

Metalloporphyrin-based metal–organic frameworks (MOFs) offer a unique platform for probing how confined environments influence porphyrin metal site structure and reactivity. The MOF series, FeCl-PCN-222 and MnCl-PCN-222, are investigated under a series of guest environments, including acetone, imidazole, pyridine, and piperidine, to demonstrate how differences in metalloporphyrin site accessibility and reactivity influence guest molecule axial ligation patterns. Fe and Mn K-edge X-ray absorption spectroscopy (XAS) is employed to characterize the local coordination, oxidation state, and electronic structure of the porphyrin metal centers within the frameworks under those guest environments. A set of molecular metalloporphyrin complexes with well-defined axial coordination status, oxidation, and spin states is measured for comparison and to aid interpretation of the MOF porphyrin coordination patterns. XANES pre-edge features, assigned and interpreted with the help of time-dependent density functional theory (TD-DFT) calculations based on the molecular models, reveal that MOF metalloporphyrin geometries deviate from the molecular analogues for some metalloporphyrin linker/guest combinations. These findings are discussed in the context of framework-imposed pore size restrictions and differences in both porphyrin ring distortion and initial chloride ligand distribution, with the latter two factors further supported by X-ray crystallographic results.

## Graphical abstract

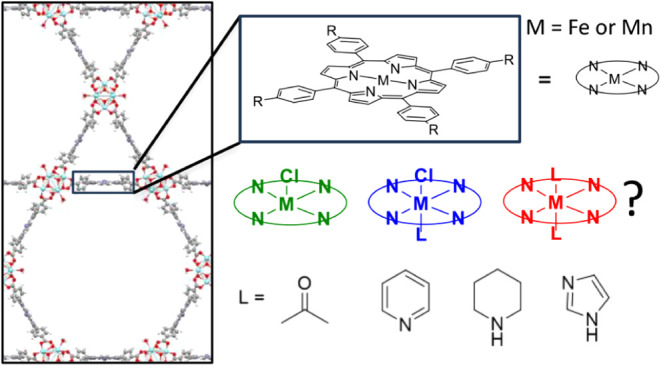

## Introduction

Incorporation of metalloporphyrin macrocycles into synthetic porous solid-state matrices, such as metal–organic frameworks (MOFs), can produce biomimetic materials with similar small-molecule activation and catalysis functionality as found in their native protein environments.^1–3^ Metalloporphyrin-based MOFs are self-assembled 3D crystalline networks of metal-oxo clusters connected through coordination bonds with metalloporphyrin linker molecules.^3–13^ With permanent microporosity upon solvent removal and exchange, these robust structures offer guest species access to the porphyrin metal centers that comprise the framework pore walls. Their host–guest interactions have been utilized for catalytic reactions such as olefin epoxidation, biomimetic oxidation, and CO_2_ reduction.^5,8,9,14–18^ In most examples, the intended role of the framework is simply to isolate the high density of metalloporphyrin linkers, preventing dimerization pathways while allowing axial interaction with potential reactant guest species within the pores. Exploiting the influence of framework-imposed constraints on the axial ligation and reactivity of the metalloporphyrin linkers would provide additional control over the catalytic behavior of these materials.

In previous studies, we probed a series of metalloporphyrin-based MOFs, namely PCN-222 ^5^ and PCN-224,^7^ under different coordinating guest molecule environments to reveal the role of pore size restrictions in dictating axial ligation behavior.^19–21^ For example, upon the introduction of electronically reducing nitrogenous base guest species, such as piperidine, we found that the small triangular pores of PCN-222 restricted the axial ligation of this coordinating species, leading to a 2:1 ratio of 5- to 6-coordinate iron­(II) porphyrin sites. The larger pore size of PCN-224, however, posed minimal restriction on piperidine axial ligation, leading to predominantly 6-coordinate (i.e., double axial ligation) iron­(II) porphyrin sites with this nitrogenous base. Notably, in these cases, the addition of piperidine guest species to iron porphyrin linkers elicited the same metal oxidation and spin-state changes as reported for other iron porphyrin complexes with analogous 5- and 6-coordinate environments (i.e., reduction to Fe^2+^ with high and low spin states, respectively). In other words, the reactivity of the iron porphyrin sites within the framework was purely dictated by the pore size restrictions of the specific MOF structures but otherwise proceeded as expected for iron porphyrin complexes in similarly restricted (or unrestricted) environments.

In this paper, we introduce some examples of metalloporphyrin MOF-coordinating guest combinations that do not necessarily follow the same reactivity patterns as reported for the analogous metalloporphyrin complexes. Combined with pore size restrictions, other structural factors, such as the intrinsic deformation of the metalloporphyrin linkers and the initial presence and pore-facing orientation of axial ligands in the native frameworks, can also dictate the metalloporphyrin coordination patterns that emerge upon the introduction of different guest species. These additional structural factors are effectively determined by the nature of the porphyrin-bound metal ions. For example, the d^4^ electron configuration of high-spin (HS) Mn­(III) in some porphyrin coordination environments can lead to axial bond elongation due to Jahn–Teller distortion whereas HS Fe­(III) with analogous coordination does not experience this symmetry breaking effect.^22^ At the same time, the electron configuration of HS Fe­(III) (d^5^) involves occupation of the d_x2‑y2_ orbital leading to σ-antibonding interactions with the porphyrin macrocycle and substantial out-of-plane metal displacement—an effect not found in Mn­(III) porphyrin complexes, where the d_x2‑y2_ orbital remains unoccupied.^23^ As linkers in some framework environments, these metalloporphyrins and their metal-specific coordination differences can drive axial ligation patterns.

Focusing exclusively on the PCN-222 framework, this study compares the Mn-porphyrin version upon the introduction of a range of guest species, including acetone, imidazole, pyridine, and piperidine, with the analogous Fe-porphyrin MOF-guest combinations. As in our previous work, we use metal K-edge X-ray spectroscopy as an element-specific, structurally, and electronically sensitive probe method for characterizing these frameworks under different guest environments. Several model metalloporphyrin complexes with known metal oxidation, spin, and axial coordination status are measured as well for comparison. We focus primarily on the XANES region of either Fe or Mn K-edge X-ray absorption spectra, as the edge and pre-edge features reveal metal oxidation, spin, and coordination geometry information. Crucially, TD-DFT XAS calculations for a range of relevant iron and manganese porphyrin models aid our interpretation of the experimental spectra of the corresponding MOF host–guest systems. Calculated XAS transitions confirm acceptor orbital parentage and predict relative energies and intensities that are consistent with the experimental pre-edge feature appearances. These computationally supported interpretations reveal unexpected porphyrin axial ligation patterns in the MOF for some metalloporphyrin linker/guest combinations. We attribute these findings to a combination of framework-imposed pore size restrictions, differences in porphyrin ring distortion, and initial metal-chloride ligand bond length and distribution, which are further supported by X-ray crystallographic results.

## Methods

### Synthesis

#### FeTPP­(THF)_2_Ag­(THF)_2_ClO_4_

The reaction was carried out using a modified literature procedure^24^ in dry THF and hexane under an N_2_ atmosphere. FeClTPP (1.00 g, 1.42 mmol) was dissolved in THF (60 mL), and AgClO_4_ (2.84 mmol) was subsequently added. The solution was boiled for 30 min while stirring at 66 °C. After cooling to room temperature, the AgCl byproduct was removed using a coarse frit funnel and Celite. 100 mL of hexane was slowly added to the THF filtrate and left to crystallize overnight at room temperature. Analysis: single-crystal XRD. Yield: 832 mg, 48.6%

#### FeTPPPy_2_ClO_4_

Pyridine and heptane were dried over molecular sieves. Following an adapted literature procedure,^24^ 30 mg of FeClTPP was added to 2 mL of pyridine (24.6 mmol). After 1 min of vigorous shaking of the reaction mixture, 16 mL of heptane was slowly added to form two layers. After 12 h, single crystals were harvested for SCXRD. Analysis: single-crystal XRD. Yield: 18 mg, 82%.

#### MnCl-PCN-222

FeCl-PCN-222 and MnCl-PCN-222 were synthesized and activated according to published procedures.^5^ Acetone, pyridine, or piperidine guests were introduced by the following procedure: 50 mg of activated MOF was soaked in 10 mL of guest under a nitrogen atmosphere for 24 h, and then the excess guest was removed by filtration. Imidazole was introduced using a published procedure.^20,25^ Retention of crystallinity and phase of each guest-loaded framework was confirmed by powder XRD (Figure S1), and the interaction of the guests with the porphyrin linker was evidenced by UV–vis diffuse reflectance spectroscopy (Figure S3).

### X-ray Absorption Spectroscopy

X-ray absorption data were collected at the Mn (6539 eV) and Fe (7111.2 eV) K-edges in transmission mode at 20ID at APS or 6-BM at NSLS-II. At both beamlines, the X-ray white beam was monochromatized by a Si(111) monochromator, and transmission mode data were collected using ion chambers filled with 100% N_2_ to measure the incident (*I*_0_), transmitted (*I*_*t*_) and reference (*I*_*r*_) beam intensity. Fe and Mn foils were used as references for energy calibration. For the reference complexes, a mixture of 20 mg of sample thoroughly ground with ∼100 mg of boron nitride was packed into Kapton tubes and heat-sealed to yield approximately one X-ray absorption length. MOF samples were thoroughly ground and used without additional dilution. Air-sensitive samples were packed in the capillary tubes in the glovebox and sealed as previously described.^20^ All data were collected at room temperature. At 20ID, damage prevention was achieved by using a previously established protocol^20^ involving a defocused X-ray spot size at the sample, with X-ray shuttering between each movement of the monochromator and linear sample translation after each scan. For measurements collected at 6-BM, while X-ray damage was not observed, samples were translated between scans to ensure a fresh irradiation spot out of an abundance of caution. Three to 6 scans were collected and averaged for each sample. Experimental spectra were processed, and linear combination fits were performed using the Demeter software package.^26^

### X-ray Emission Spectroscopy

Nonresonant Fe and Mn Kβ XES spectra were collected at the APS 20-ID beamline by binning XES spectra measured over an incident X-ray energy range spanning 800 to 840 eV above the respective absorption edge. A miniXES spectrometer,^27^ equipped with a Pilatus 100K detector and either GaP­(4,4,0) flat crystals for Mn Kβ XES or Ge­(6,2,0) flat crystals for Fe Kβ XES, was used for emission analysis. Thoroughly ground, undiluted samples were packed in 1 mm Kapton tubes that were heat-sealed prior to measurement. XES data were collected using a similar damage prevention protocol as described above. Additionally, for Mn Kβ XES, the beam was attenuated using an aluminum filter and shuttered during monochromator movements, while the acquisition time for each scan was set to 0.1 s for each energy. Data were averaged after 100 scans.

### Computational Methods

The computational models used for the iron and manganese centers were obtained either from known crystal structures^25,28–31^ or by geometry optimization using the ORCA 4.2.1 package^32^ performed with the PBE0 functional,^33^ TZVP­(−f)^34^ basis set with the auxiliary def2/J basis set,^35^ zeroth-order relativistic approximation (ZORA),^36^ and resolution of identity approximation for Coulomb and HF exchange (RIJCOSX). XAS calculations were performed using the TD-DFT approach implemented in the ORCA 4.2.1 package, employing the PBE0 functional^33^ and a split basis set consisting of the TZVP­(−f)^34^ basis set with the auxiliary def2/J basis set for all atoms except iron or manganese, which used the CP­(PPP) basis set,^37^ zeroth-order regular approximation,^36^ and RIJCOSX approximation. For the simulation of XANES spectra, the first 30 roots were selected, and transitions were restricted to those originating from the Fe or Mn 1s orbitals. Simulated spectra were generated using 1.5 eV Gaussian broadening and a universal energy shift of 14.75 eV for Fe and 22.57 eV for Mn K-edge XAS. Additionally, intensity scaling factors of 0.07899 for Fe and 0.1013 for Mn were applied for better comparison with experimental data. Calculated orbitals were visualized and rendered by using the Avogadro software package.

## Results

Previously reported^20^ PXRD, UV–vis, and XAS data for FeCl-PCN-222 loaded with acetone, imidazole, and piperidine and related Fe porphyrin reference complexes, are replotted here to allow for proper comparison with the analogous Mn versions of the MOF and TD-DFT computational results. Characterization of MnCl-PCN-222 and FeCl-PCN-222 loaded with guest species (acetone, imidazole, pyridine, or piperidine) by powder X-ray diffraction (Figure S1) confirmed the PCN-222 structure, with retained crystallinity and phase upon activation and introduction of each guest molecule environment. UV–vis diffuse reflectance spectra of these frameworks, along with several relevant Mn- and Fe-porphyrin model complexes (also shown in the Supporting Information, Figures S2–S4) display the characteristic Soret and Q-bands attributed to the metalloporphyrin linkers, with wavelength maxima summarized in Tables S1 through S4. For manganese porphyrin complexes, axial ligation with imidazole, pyridine, or piperidine tends to blue-shift both the Soret and Q bands.^31,38,39^ A similar trend is observed for the MnPCN-222 MOFs under analogous axially coordinating guest environments, albeit with more subtle spectral shifts. For the iron porphyrin reference complexes, axial ligation can be accompanied by metal center oxidation and/or spin-state changes, depending on the ligand. This, in turn, leads to more pronounced spectral differences, particularly in the shift and number of Q bands. For Fe-PCN-222 under imidazole and piperidine guest environments, their diffuse reflectance spectra follow the trend established by the respective reference complexes (see ref. 20 Figure 2). For pyridine-loaded FeCl-PCN-222, however, the spectrum deviates from the FeTPPPy_2_ClO_4_ spectrum as well as that of the activated FeCl-PCN-222 framework with no axially coordinated species, indicating a more complex axial ligation behavior. For weakly interacting guests such as acetone, for which molecular analogues do not exist, UV–vis diffuse reflectance spectra reveal the binding behavior afforded by the framework environment. For MnCl-PCN-222, a red shift of Soret and Q bands indicates interaction with this guest molecule, while no changes are observed in the Fe version of the MOF (see Figure S4 of ref. 20).

### Crystallography

The crystal structure obtained for the MnCl-PCN-222 MOF is shown in the Supporting Information. Figure 1 depicts the structure of the metalloporphyrin macrocycle linkers of this MOF in comparison with that of the previously reported Fe version^5^ and corresponding metalloporphyrin complexes.^25,31^ The porphyrin macrocycle of FeClTPP and FeCl-PCN-222 shows similar distortions. The out-of-plane distance of Fe from the porphyrin macrocycle in FeCl-PCN-222 and the complex is 0.309 Å and 0.372 Å, respectively. Furthermore, the porphyrin macrocycle itself in both the Fe complex and MOF displays similar planarity. In contrast, comparing MnCl-PCN-222 with the MnClTPP complex reveals stark differences between their metalloporphyrin macrocycle structures. Most notably, the porphyrin macrocycle is significantly ruffled in the Mn complex compared to the more planar macrocycle in MnCl-PCN-222, with the latter more closely mimicking the Fe version of this MOF. The Mn metal center in the MnCl-PCN-222 structure displays an out-of-plane distance of 0.154 Å compared to the 0.257 Å distance found in the MnClTPP complex structure. This apparent reduction in out-of-plane distance, in part, reflects the distribution of the axial Cl orientation in this framework, with the structure revealing that 72% of the Cl population is coordinated on the triangular pore-facing side and 28% is located on the large pore-facing side of the porphyrin linkers. This distribution of axial chloride orientation, combined with the change in ring distortion compared to the Mn complex, suggests that its axial ligation behavior and overall reactivity, in general, may be different from that of the native complex.

**Figure 1:**
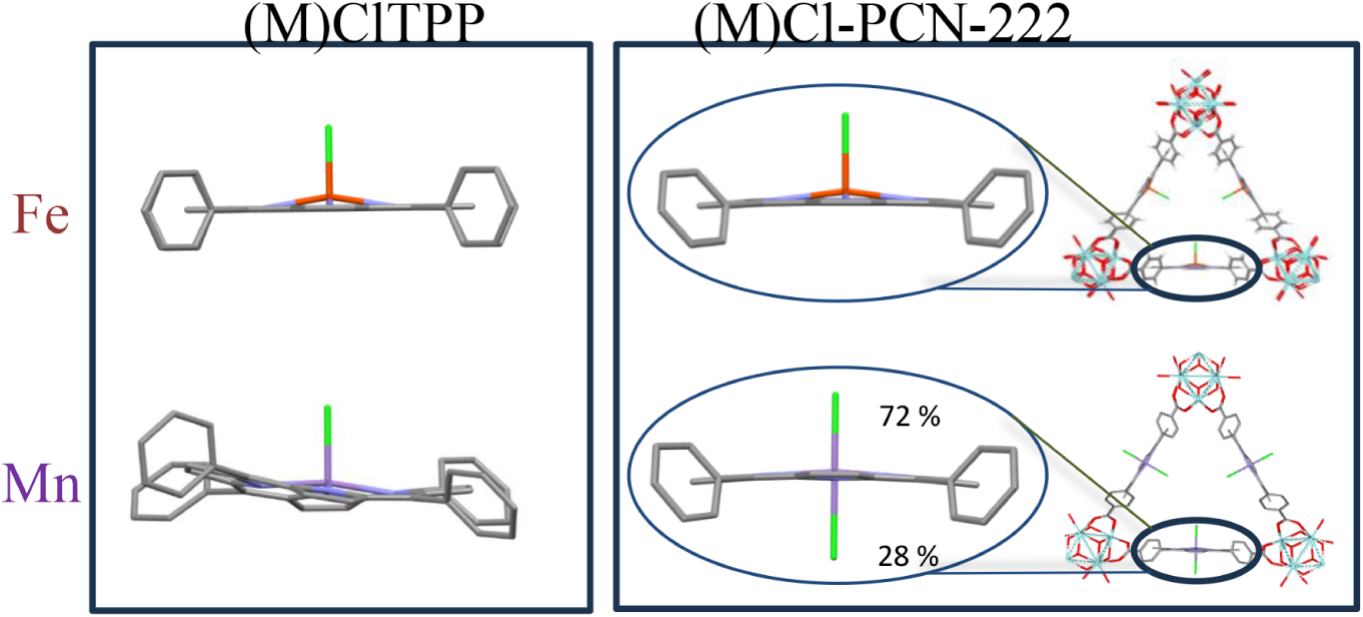
Crystal structures depicting the deviation in planarity of the metalloporphyrin ring in (top row) FeClTPP (structure obtained from ref. 40) and FeCl-PCN-222 (structure obtained from ref .^5^); (bottom row) MnClTPP (structure obtained from ref. 31) and MnCl-PCN-222 (structure obtained in this work). Percentages indicate the distribution of Cl axial ligand orientation obtained from the crystal structure: 72% triangular pore-facing and 28% hexagonal pore-facing Cl.

### X-ray Spectroscopy

XANES spectra collected for all MOFs and reference complexes at the Mn and Fe K-edge are shown in Figures S5–S8, with the spectra plotted in Figures 2–6 highlighting their corresponding pre-edge regions. Experimental spectra obtained for most of the Fe systems (FeClTPP, FeTPPIm_2_Cl, FeTPPPip_2_, and FeCl-PCN222 with acetone, imidazole, and piperidine guest environments) previously reported in ref. 20 (Figures 4 and S7) are included here for comparison with the Mn analogues, TD-DFT-calculated spectra, and across the full range of axially coordinating guest species, including pyridine. The rising edge energy of each spectrum was used to assess the metal oxidation state, while the 1s → 3d pre-edge features provide local coordination geometry in addition to oxidation and spin state information. Linear combination fits of the MnCl-PCN222-Im, MnCl-PCN222-Py, and MnCl-PCN222-Pip experimental spectra were performed using MnCl-PCN222-act and relevant reference complex spectral components. For FeCl-PCN222-Py, fits were performed using FeClTPP, FeTPPPy_2_ClO_4_, and FeTPP­(THF)_2_ClO_4_ spectral components, as discussed below. The experimental data, fits, and residuals are shown in Figure S9 and in Tables S5 and S6. The shape and intensity of the pre-edge features vary with the metal and guest environment and can be interpreted with the support of TD-DFT calculations, as discussed in detail below. Nonresonant Mn and Fe Kβ XES measurements collected for the PCN-222 MOFs under select guest environments and relevant metalloporphyrin reference complexes with known metal spin states are shown in Figure S13. The data were analyzed using the integrated area under difference spectra (IAD) method^19,41–43^ (Figure S14, Table S7) to assess the average spin state of the porphyrin metals in the MOF environments. Table S8 summarizes these XAS and XES results for each system, along with the corresponding porphyrin metal oxidation and spin states.

**Figure 2:**
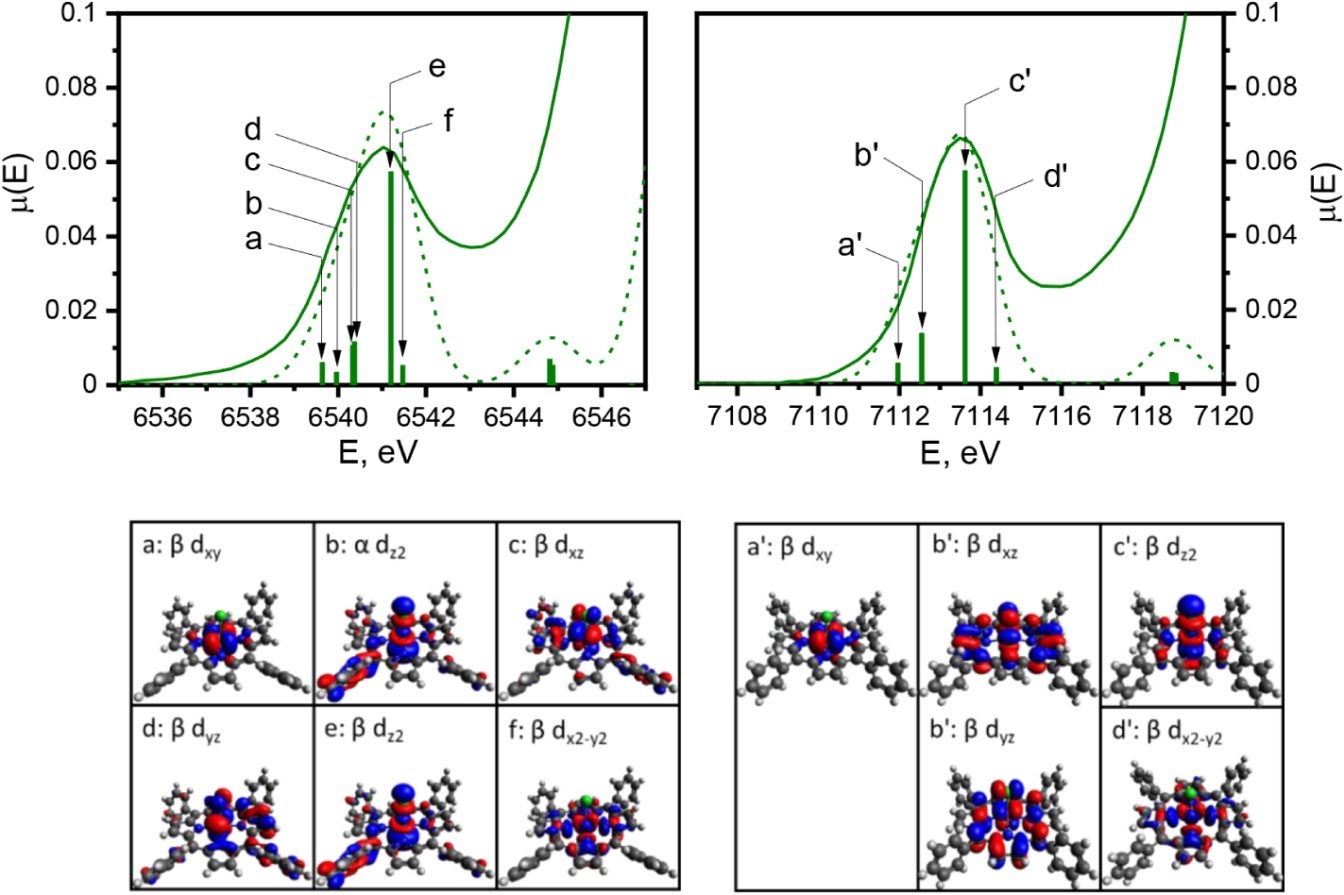
Top: MnClTPP and FeClTPP; solid line – experimental XANES, dashed line – calculated XANES, vertical bars – calculated transitions; Bottom: major contributing acceptor orbitals associated with pre-edge transitions.

### Computational Results

Representative calculated XAS spectra with 1.5 eV applied broadening and underlying transitions are plotted along with the relevant experimental spectra for the MnClTPP and FeClTPP reference complexes in Figure 2. Images of the dominant acceptor orbitals associated with these transitions are shown below each set of spectra. The calculated spectra for the rest of the Fe and Mn porphyrin models are shown in Figures 3–6 and the full set of calculated spectra and underlying transitions is shown in Figures S15–S29. The complete list of transitions with dominant acceptor orbital parentage for each model can be found in Tables S9–S23. These calculation results and corresponding transition assignments align with well-established ligand field theory trends for metal centers in approximate octahedral and square pyramidal coordination environments. For penta-coordinated metal centers, such as those found in MnClTPP and FeClTPP, the calculated pre-edge feature has increased intensity. For this coordination geometry, the underlying transition is dominated by 1s → 3d_z2_ character but is partially dipole-allowed due to the 4p mixing associated with the lower symmetry. Hexa-coordinated metal centers with one chlorine (e.g., MnClTPPPy) have decreased intensity with shoulders at lower energies; however, the most intense transition is still dominated by the 1s → 3d_z2_ transition. The lowest intensity pre-edge features are found for symmetric hexa-coordinated centers with approximate D_4h_ symmetry. While high-spin (HS) hexa-coordinated Mn­(III) and Fe­(III) systems (e.g., MnTPPPip_2_Cl and FeTPP­(THF)_2_ClO_4_) yield a calculated pre-edge that is split into two features (arising from the “t_2g_” “e_g_” d-orbital splitting), low-spin (LS) Fe­(II) octahedral environments (e.g., FeTPPPy_2_) predict a single pre-edge peak since t_2g_ orbitals are fully occupied and the only possible transition is 1s → 3d­(e_g_). LS Fe­(III) metal centers in this coordination geometry (such as FeTPPIm_2_Cl) have a low intensity pre-edge feature with a shoulder around 7111.0 eV corresponding to the 1s → 3d­(t_2g_) transition.

**Figure 3:**
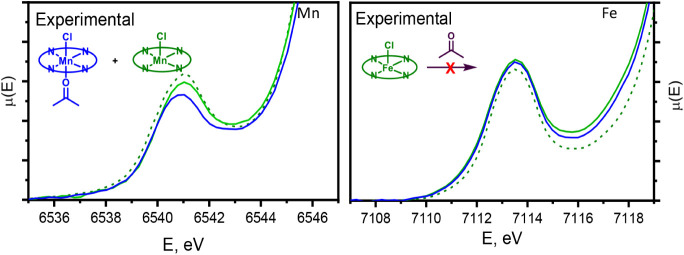
Experimental XAS spectra of, left: MnClTPP (dark green, dashed line), MnCl-PCN-222-act (light green), MnCl-PCN-222-acetone (blue), and right: FeClTPP (dark green, dashed line), FeCl-PCN-222-activated (light green), and FeCl-PCN-222-acetone (blue).

## Discussion

Spectroscopic characterization of the Mn- and Fe-porphyrin-based MOFs under different guest environments, in comparison with molecular analogues, reveals the influence of the framework-imposed pore size restrictions and porphyrin ring distortions in dictating axial ligation patterns in these materials. Here, we compare these analyses in terms of the electronic and geometric structures of the metalloporphyrin sites under the different guest environments measured, and the role of the frameworks in directing the axial ligation behavior for the two metals. UV–vis DR provides some hints of axial coordination (along with metal oxidation/spin state) status under the different guest environments. While the porphyrin Soret and Q-band sensitivity to axial ligation status is well-established in both molecular and polymeric environments,^21,44^ it is not universally sensitive for all metals, as discussed in the results section. Stronger evidence of guest binding at the metalloporphyrin linker sites is provided by XANES characterization, in particular the pre-edge region, with well-established sensitivity to metal coordination geometry.^45^ On a qualitative level, increased pre-edge feature intensity indicates a decrease in the symmetry of the metal center, since the resulting metal 4p contribution enhances the dipole-allowed character of the underlying transitions. Furthermore, the splitting pattern allows an estimation of the type of transitions and the spin state of the metal center. In general, the pre-edge XANES spectrum of each MOF/guest system is compared with that of the corresponding metalloporphyrin reference complexes with known local geometry to provide insight into the coordination environment of the metalloporphyrin center in the framework under the analogous guest environment. In some cases, linear combination fitting of the MOF XAS spectra using the relevant metalloporphyrin reference complex spectra helped establish the relative contributions of those coordination environments. Using molecular systems with known coordination environments and metal oxidation and spin states as models, TD-DFT calculations afford a more detailed interpretation of the pre-edge feature assignment and the associated metal coordination environments contributing to the spectra.

### Acetone

As a weakly coordinating solvent, we did not expect much interaction between acetone, as a guest species, and the metalloporphyrin sites in the MOF environment. While FeCl-PCN-222 behaved as expected, MnCl-PCN-222 showed a weak binding behavior. In situ diffuse reflection measurements collected upon activation of the acetone-loaded MOFs (Figure S4) displayed a red shift of the Soret band from 481 to 485 nm for the MnCl-PCN-222 MOF, but no spectral changes for FeCl-PCN-222. These observations suggest that acetone interacts with the metalloporphyrin sites in MnCl-PCN-222 but not in FeCl-PCN-222. XANES measurements of these MOFs provided more solid evidence for this trend. As illustrated in Figure 3, the pre-edge features observed in the spectra of MnClTPP and MnCl-PCN-222-act have very similar shapes and intensities, with a maximum around 6541 eV. Under an acetone guest environment, however, the MnCl-PCN-222-ace spectrum displays comparatively less intensity, indicating weak interaction of the acetone guest with the Mn porphyrin linkers of the MOF. The edge positions for both complexes and MOFs are very similar, with energies around 6546.2 eV, confirming the presence of HS Mn­(III) centers in each case. In contrast, the activated FeCl-PCN-222, FeCl-PCN-222-ace, and FeClTPP display nearly identical XANES spectra, with not only identical edge energy positions for the complexes and MOFs at 7122.5 eV, confirming the presence of HS Fe­(III) sites in each case, but also the same pre-edge feature energy and intensities as well, denoting minimal interaction between the Fe centers and the acetone guest species (Figure 3).

The divergent behavior observed between the Mn- and Fe-porphyrin-based MOFs in the presence of acetone may be explained by the structural differences of the porphyrin metal centers. In metalloporphyrin complexes, the M-N bonds are known to be longer in Fe­(III) porphyrin compared to Mn­(III) porphyrin, due to differences in d-orbital occupancy between the two HS metal ions.^23^ This difference in equatorial coordination leads to higher planarity of the manganese porphyrin core and a more accessible sixth coordination position compared to analogous Fe­(III) porphyrin environments, in which the metal center lies out of the macrocycle plane, as illustrated in Figure 1.

### Imidazole

As a strongly coordinating ligand, imidazole is expected to readily bind to both Fe and Mn porphyrin sites when it is incorporated as a guest species in these MOFs. Model complexes with these axial ligands derived from MnClTPP or FeClTPP display hexacoordination, with one imidazole located at the open axial site and the other replacing the chloride ion on the metal center. In the process, as established by literature precedent,^25,46–48^ while Mn retains its high-spin state, Fe undergoes a high- to low-spin-state transition. UV–vis titration studies of the MnClTPP complex with imidazole revealed a two-step reaction wherein the MnClTPPIm is formed first, followed by replacement of the chloride ligand with a second imidazole molecule.^31^ XANES measurements provide insight into how the framework environment leads to subtle differences in imidazole coordination at the metalloporphyrin coordination site within these MOFs. For MnCl-PCN-222-Im, we observed an increased intensity of the pre-edge feature around 6541 eV, indicating contributions from different coordination environments in the MOF compared to the symmetrically coordinated MnTPPIm_2_Cl reference complex. The linear combination fit of this spectrum, using the MnCl-PCN-222-act and MnTPPIm_2_Cl experimental spectra (Figure 4a), bore a composition of 27% and 73%, respectively. This ratio is strikingly similar to the distribution of the chlorine atoms in the MOF revealed by crystallography (Figure 1), where, statistically, 28% of the chlorine atoms point to the large pores and 72% point to the small pores. Together, this evidence suggests incomplete imidazole coordination of the Mn sites in MnCl-PCN-222-Im and that the pattern of Cl ligand replacement is linked to their pore-facing location in terms of accessibility.

The calculated spectra using relevant porphyrin models and their linear combinations (Figures 4b and S9) allow us to further consider contributions from different imidazole coordination environments, including those that are not experimentally accessible as model complexes. The linear combination of the MnClTPP and MnTPPIm_2_Cl calculated spectra correlates well with the shape and intensity of the analogous experimental linear combination fit using the same 27% and 73% compositions, respectively (Figure 4b). Replacing the MnClTPP calculated spectrum with that of the MnClTPPIm model, where the Mn center has a hexa-coordinated environment with one coordinated imidazole and a chlorine atom, leads to a significantly less intense pre-edge when this same composition ratio is used (Figure S10). A composition of 65% MnClTPPIm and 35% MnTPPIm_2_Cl in the linear combination spectrum (also shown in Figure S10) is needed to achieve the appropriate peak intensity, although the intensity of the lower-energy side of the feature falls short. While we cannot rule out some contribution of this MnClTPPIm coordination, the closer pre-edge feature intensity and shape match, achieved using the MnClTPP and MnTPPIm_2_Cl models, suggest that these are the dominant environments contributing to the overall spectrum. Furthermore, the similar composition ratio to that of chlorine atom orientations in the native MOF structure supports our conclusion that the initial distribution of the chlorine atoms in the MOF ultimately restricts the double ligation of imidazole guests at the Mn porphyrin linker sites.

**Figure 4:**
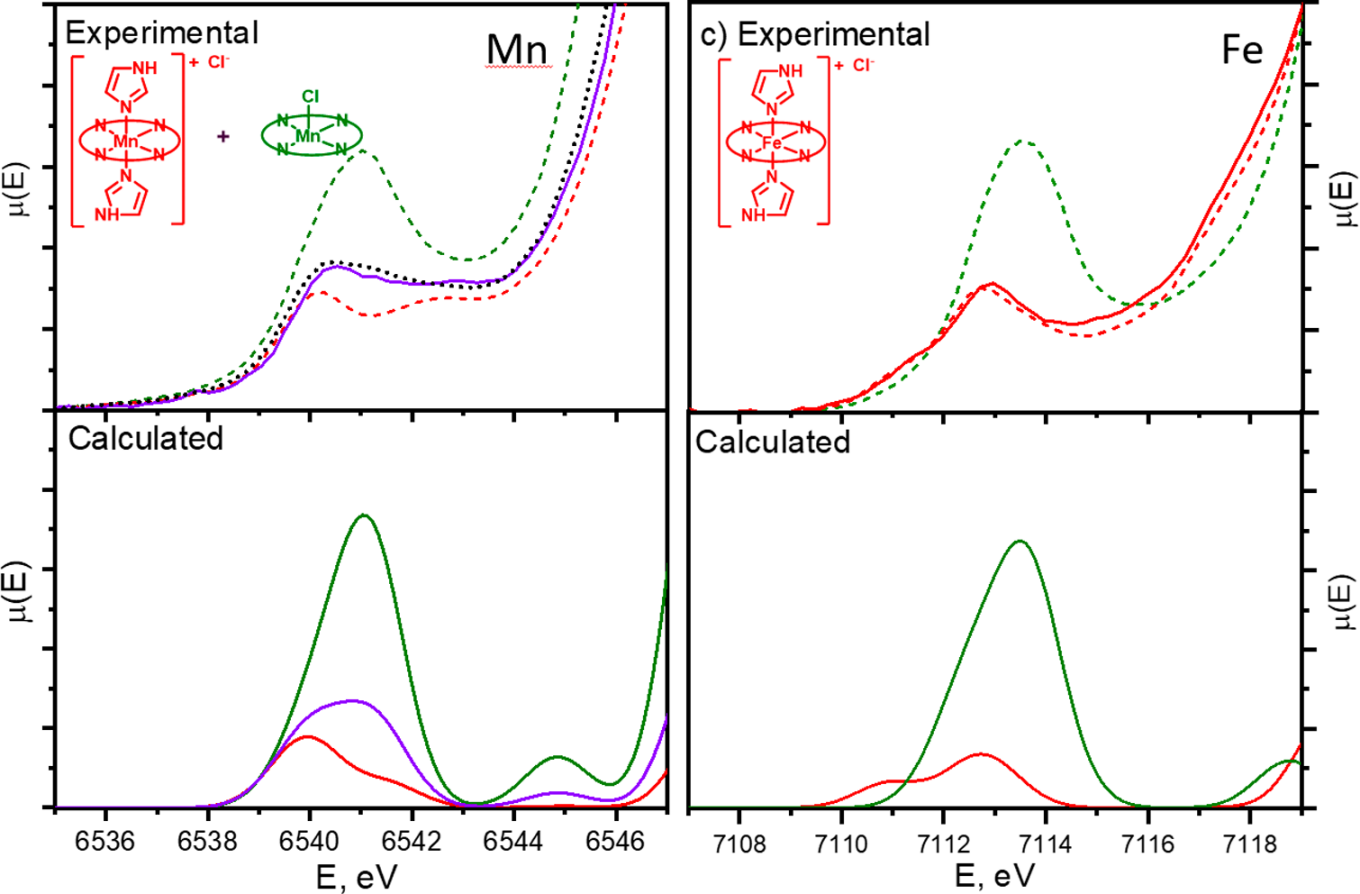
a) Experimental XAS spectra for MnCl-PCN-222-Im (purple), MnClTPP (dashed green), MnTPPIm_2_Cl (dashed red), linear combination of MnClTPP (26.7%) and MnTPPIm_2_Cl (73.3%) (dotted black). b) Calculated XAS for MnClTPP (green), MnTPPIm_2_Cl (red), and linear combination of MnClTPP (26.7%) and MnTPPIm_2_Cl (73.3%) (solid purple). c) Experimental XAS spectra for FeCl-PCN-222-Im (red), FeClTPP (dashed green), FeTPPIm_2_Cl (dashed red). d) Calculated XAS for FeClTPP (green), FeTPPIm_2_Cl (red).^31^

Unlike the Mn framework, FeCl-PCN-222-Im yields experimental pre-edge features at 7112.5 eV with a shoulder around 7111 eV, which are almost identical in intensity to those of the doubly ligated reference complex, FeTPPIm_2_Cl (Figure 4c). Comparison of these data confirms that all metal centers in FeCl-PCN-222-Im are hexacoordinated with two imidazole molecules attached to the iron. Furthermore, the pre-edge feature shape and rising edge energy of the XANES spectra for the MOF and the complex indicate the presence of LS Fe­(III) centers. The calculated spectrum for the LS Fe­(III) metal center in this imidazole coordination environment matches the shape and intensity of the pre-edge feature observed experimentally. As confirmed by the acceptor orbital parentage for the underlying transitions (Table S17), the shoulder feature arises from a transition from 1s to d_t2g_ orbitals (assuming approximate octahedral geometry). The intensity of the peak at 7112.5 eV arises from 1s to d_eg_ transitions where the degeneracy is relaxed, and the individual 1s to d_z2_ and d_x2‑y2_ transitions occur at slightly different energies (see Figure S23).

The divergent axial binding behavior of the Mn and Fe porphyrin sites with imidazole in the framework environment can be traced to the different space requirements of the metal centers for accommodating the M-Im axial bond. Reference complexes FeTPPIm_2_Cl and MnTPPIm_2_Cl shed light on the divergent coordination patterns of imidazole in the PCN-222 MOFs. While manganese does not change its spin state upon interaction with imidazole, iron undergoes a high- to low-spin transition, which alters the ionic radii. Due to the smaller ionic radius of LS Fe­(III), the Fe-Im bond length is only 1.977 Å in FeTPPIm_2_Cl, while the larger HS Mn­(III) ionic radius in MnTPPIm_2_Cl results in a corresponding axial bond length of 2.284 Å. The longer bond length effectively limits the number of imidazole molecules that can occupy the triangular pore-facing porphyrin sites in MnCl-PCN-222-Im.

### Pyridine

While pyridine is a strongly coordinating ligand, model complexes derived from MnClTPP or FeClTPP upon pyridine exposure demonstrate different axial-ligation behaviors. In the former case, only one molecule of pyridine coordinates, forming the hexacoordinate MnClTPPPy complex.^31^ Interaction of pyridine with FeClTPP, however, displaces the Cl ligand and leads to the formation of FeTPPPy_2_^+^, a hexacoordinated species with two axial pyridine ligands^49,50^ and an accompanying high-to-low spin state change of the Fe­(III) centers.

Including these metalloporphyrin sites within the MOF environment leads to different pyridine axial ligation outcomes than those of their molecular analogues, as revealed by XANES characterization. For MnCl-PCN-222-Py, we observe similar behavior to the imidazole system described previously, in that the pre-edge feature suggests a combination of 5- and 6-coordinate axial ligation environments. In this case, the hexacoordination pattern likely follows that of the MnClTPPPy reference complex. As shown in Figure 5a, the linear combination fit, using the MnCl-PCN-222-act and MnClTPPPy spectra, is a good match and yields a distribution of 22% and 78% for these respective penta- and hexacoordinate porphyrin model environments. Again, this ratio tracks the large versus small pore-facing distribution of chloride ligands in the MOF prior to guest incorporation.

**Figure 5:**
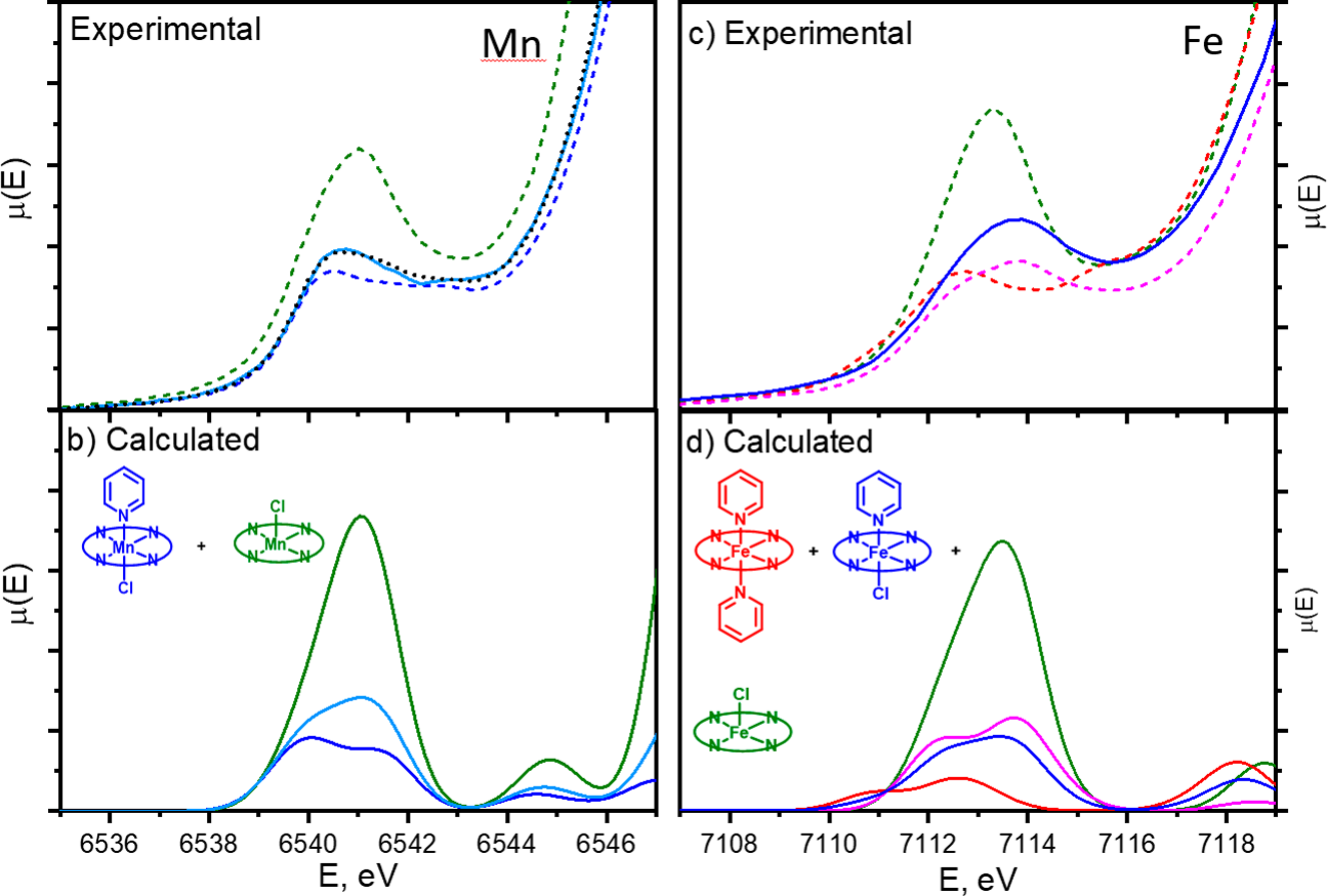
a) Experimental XAS spectra for MnCl-PCN-222-Py (light blue), MnClTPP (dashed green), MnClTPPPy (dashed blue), linear combination of MnClTPP (21.6%) and MnClTPPPy (78.4%) (dotted black). b) Calculated XAS for MnClTPP (green), MnClTPPPy (blue), linear combination of MnClTPP (21.6%) and MnTPPPy (78.4%) (light blue). c) Experimental XAS spectra for FeCl-PCN-222-Py (blue), FeClTPP (dashed green), FeTPPPy_2_Cl (dashed red), FeTPP­(THF)_2_ClO_4_ (dashed magenta). d) Calculated XAS for FeClTPP (green), FeTPPPy_2_ClO_4_ (red), FeClTPPPy (magenta), linear combination spectrum (blue) (see text for details).

The linear combination of calculated spectra using relevant porphyrin models further supports this distribution of coordination environments. The calculated spectral components (Figure 5b) correlate well with the experimental results for the corresponding reference complexes. The MnClTPPPy spectrum displays a lower-intensity pre-edge compared to that of the pentacoordinated MnClTPP complex, as observed experimentally, due to the higher symmetry of its metal center. Compared to the calculated spectrum, the splitting of this feature is not as well resolved in the experimental data, but increased intensity around 6542 eV points to the presence of additional transitions in this energy range. A linear combination of these calculated spectra shows strong agreement with the one derived from the analogous experimental spectra, further supporting the dual presence of both MnClTPP and MnClTPPPy coordination environments in the MOF at this ratio.

The framework environment similarly influences metalloporphyrin axial ligation in FeCl-PCN-222 upon the introduction of pyridine guests, but with the added complexity of potential spin-state changes of the Fe centers. The XANES spectrum of FeCl-PCN-222-Py is compared in Figure 5c to those of the pertinent reference complexes that mimic the likely metal axial ligation and spin environment contributions. As a hexacoordinated LS Fe­(III) reference, FeTPPPy_2_ClO_4_ displays a low-intensity pre-edge feature with an unresolved shoulder on the lower-energy side, similar to that observed for FeIm_2_TPP, the reference complex with analogous metal spin and coordination geometry. FeTPP­(THF)_2_ClO_4_, as a synthetically accessible, hexacoordinated, *high-spin* Fe­(III) reference, presents a broad, unresolved pre-edge feature with similar low intensity but notably higher peak energy compared to the LS Fe­(III) reference. As expected, the pre-edge features for both references have lower intensity than that of the pentacoordinated LS Fe­(III) complex, FeClTPP. A qualitative spectral comparison between FeCl-PCN-222-Py and these reference complexes suggests that the majority of the Fe porphyrin sites within the MOF contain hexacoordinated metal centers, while a smaller fraction retains pentacoordination status, likely with chloride axial ligation. Furthermore, the pre-edge and edge energy shift comparisons (Figure S12) indicate a mixture of both HS and LS Fe porphyrin centers.

To better quantify this distribution of Fe coordination sites, we performed linear combination fits of the FeCl-PCN-222-Py spectrum using experimental spectra of (a) FeClTPP, (b) FeTPP­(THF)_2_ClO_4_, and (c) FeTPPPy_2_ClO_4_, as representative penta-coordinated HS Fe­(III), hexacoordinated HS Fe­(III), and hexacoordinated LS Fe­(III) components, respectively. These linear combination fits are shown in Figure S12. Targeting the edge region, the fit yields an a:b:c ratio of 1:3.37:4.32, i.e., almost 50% of the Fe­(III) centers are low spin. When the fit is limited to the pre-edge region, a ratio of 1:1.41:0.75 is obtained, implying a more limited LS Fe­(III) contribution on the order of 25%. However, this fit yields a higher R-factor (Table S6).

To further assess the distribution of Fe sites in FeCl-PCN-222-Py, we turned to Fe Kβ XES measurements and subsequent IAD analysis as a spin-sensitive probe.^20,41^ Using the XES spectra measured for Fe­(III)­ClTPP (S = 5/2), Fe­(II)­TPPPy_2_ (S = 0), and Fe­(III)­TPPIm_2_Cl (S = 1/2) complexes as known spin state references (Figure S13), the IAD analysis of the FeCl-PCN-222-Py XES spectrum yields an effective total spin of S = 1.55. This value is consistent with equal contributions of both HS (S = 5/2) and LS (S = 1/2) Fe­(III) sites, affirming the near 50/50 HS/LS distribution established through the linear combination fit of the XANES edge.

TD-DFT calculations further support these relative coordination environment contributions. The calculated spectra for the reference complexes that model the proposed Fe coordination and spin-state combinations follow the trends observed across the measured experimental spectra. The FeTPPPy_2_Cl calculated spectrum closely resembles that of FeTPPIm_2_Cl, since both complexes feature symmetrically coordinated, LS Fe­(III) centers. FeClTPP, as previously discussed, presents a more intense pre-edge due to the lower-symmetry monoaxial ligation environment. The calculated spectrum of the hexacoordinated HS Fe­(III) complex, FeClTPPPy, displays a broad pre-edge feature that resembles that of the experimentally measured FeTPP­(THF)_2_ClO_4_ reference, with analogous axial ligation and spin-state environment. The linear combination spectrum derived using these three components, with the same ratio as applied in the experimental fit, matches the trend observed for the FeCl-PCN-222-Py spectrum, further supporting the relative contributions of these proposed coordination environments in the MOF with pyridine guests.

In summary, upon the introduction of pyridine to MnCl-PCN-222, a hexacoordination scenario unfolds, where only one pyridine molecule coordinates to the Mn porphyrin linker sites, following the ligation behavior of the MnClTPPPy complex. However, some metalloporphyrin sites in the framework remain unsaturated in this guest environment, owing to the bulkier nature of the pyridine molecule (compared to other nitrogenous bases like imidazole), the comparatively elongated Mn–N­(pyridine) bond, and the initial chloride ligand orientation distribution in the framework, which restricts access to the small pore-facing axial binding sites. Furthermore, the enforced planarity of the metalloporphyrin linkers by the framework structure may also lead to incomplete axial ligation of pyridine guests. Unlike Mn, Fe porphyrin, upon coordination with pyridine, undergoes an HS to-LS transition, resulting in a significantly shorter M–N­(pyridine) bond length compared to the Mn case. Despite the propensity of Fe porphyrin to undergo double axial ligation with pyridine, the large size of this guest limits the number that can displace the Cl ligands in the small pores. These restrictions lead to complex coordination behavior, where approximately 50% of the centers are LS hexacoordinated with two pyridine molecules coordinated.

### Piperidine

Piperidine stands out from the other axially coordinating nitrogenous base guests in the series measured due to its aliphatic nature and propensity to serve as a reducing ligand. Both Fe and Mn porphyrin complexes form hexacoordinated complexes with two axial piperidine ligands, replacing the single chloride ligand in the process. Under an inert atmosphere, piperidine axial coordination is accompanied by metal site reduction from the +3 to +2 oxidation state. While FeTPPPip_2_ demonstrates reasonable stability in the solid state following reduction, the reduced complex of Mn readily reoxidizes upon exposure to air. In the presence of oxygen, manganese porphyrin forms a hexacoordinated Mn­(III)­TPPPip_2_Cl complex.^31^ Iron porphyrin, however, does not form the corresponding axially coordinated Fe­(III) complex and instead converts into the μ-oxo dimer.^51^

Piperidine interaction with these metalloporphyrin sites in the MOF environment deviates from that of their molecular counterparts, as documented through XANES characterization. The MnCl-PCN-222-Pip spectrum exhibits increased pre-edge intensity with the maximum around 6541 eV compared to that of the MnTPPPip_2_Cl complex, which suggests contributions from lower-symmetry coordination environments in the MOF. As shown in Figure 6a, the linear combination fit of the piperidine-treated MOF spectrum, using the experimental spectra of MnCl-PCN-222-act and MnTPPPip_2_Cl as model coordination environments, yields a 55% to 45% distribution ratio. This Mn-porphyrin speciation does not follow the initial chloride ligand distribution ratio, as previously described for the other coordinating nitrogenous base guests in this series -a deviation likely attributed to the larger size of piperidine guests and the longer Mn-L bond length. Furthermore, the Mn porphyrin center can potentially oxidize the piperidine and lead to the formation of the bulky imine trimer, which is known to block the pores and prevent coordination.^20^

**Figure 6:**
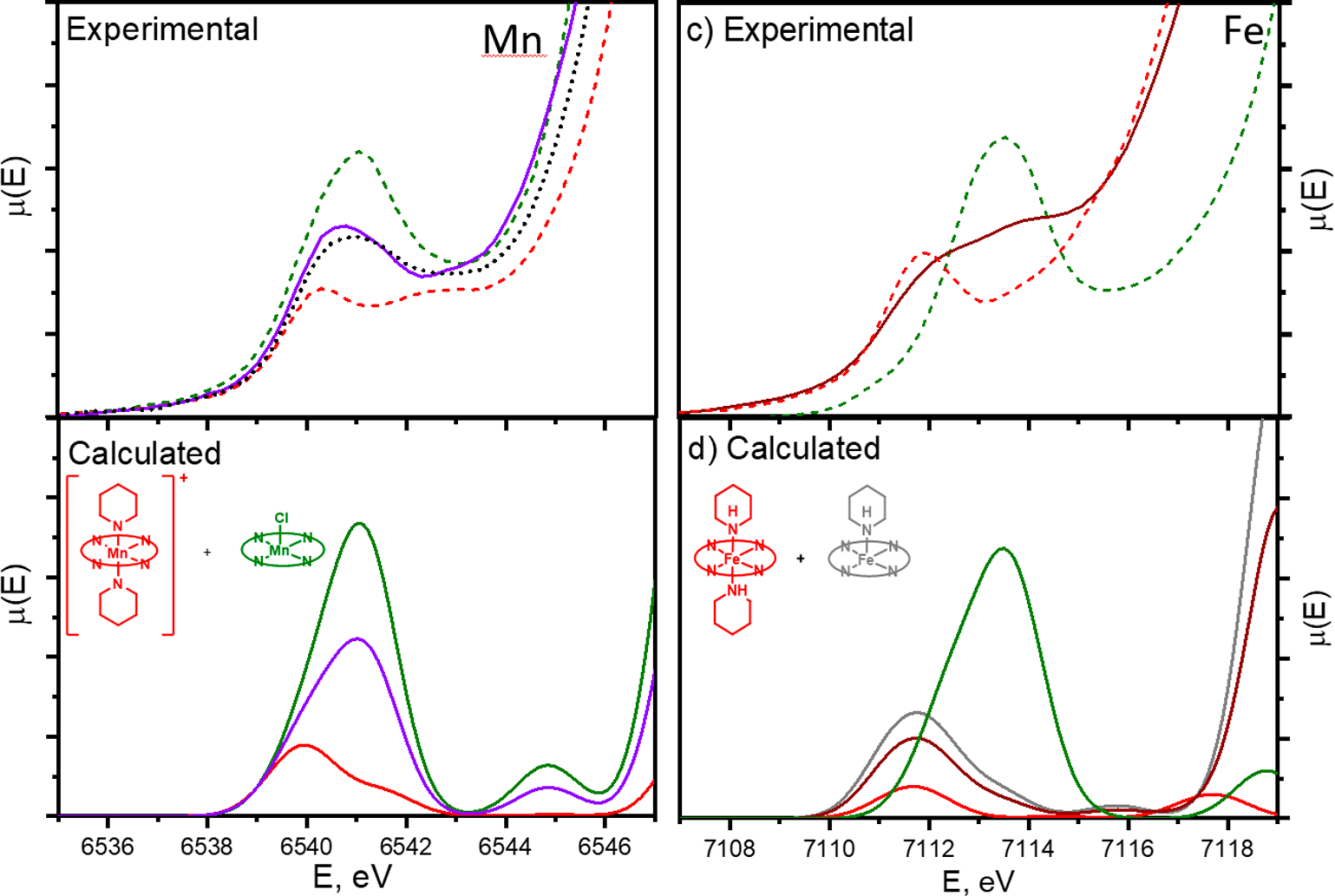
a) Experimental XAS spectra for MnCl-PCN-222-Pip (violet), MnClTPP (dashed green), MnTPPPip_2_Cl (dashed red), linear combination of MnClTPP (55.3%) and MnTPPPip_2_Cl (44.7%) (dotted black). b) Calculated XAS for MnClTPP (green), MnTPPPip_2_Cl (red), and linear combination of these calculated spectra: MnClTPP (55.3%) and MnTPPPip_2_Cl (44.7%) (purple); c) Experimental XAS spectra for Fe-PCN-222-Pip (wine), FeClTPP (dashed green), FeTPPPip_2_ (dashed red). d) Calculated XAS for FeClTPP (green) (included for reference), FeTPPPip_2_ (red), FeTPPPip (gray), linear combination of FeTPPPip (66%) and FeTPPPip_2_ (33%) calculated spectra (wine).

To exclude any significant contribution of the heteroaxially ligated MnClTPPPip coordination environment, we evaluated the TD-DFT-calculated spectrum of this molecule in comparison to those of the other model complexes, MnClTPP and MnTPPPip_2_Cl. While the calculated XAS spectrum of MnClTPPPip displays a pre-edge intensity that is similar to the linear combination of the MnClTPP and MnTPPPip_2_Cl calculated spectra (Figure S10), the maximum is shifted to higher energy (6541 eV) and does not correlate well with the trend observed experimentally.

Fe-PCN-222-Pip exhibits an XANES spectrum substantially different from those of either the FeClTPP or the FeTPPPip_2_ reference complexes (Figure 6c). Its pre-edge feature consists of two poorly resolved peaks at 7112.0 and 7113.2 eV. Our previous studies of this MOF system using XAS, XES, and Raman characterization^19^ revealed a mixture of penta-coordinated HS Fe­(II) and hexa-coordinated LS Fe­(II) porphyrin metal centers in a 2:1 ratio. TD-DFT calculations of the FeTPPPip_2_ LS and FeTPPPip HS Fe­(II) complexes presented here (Figure 6d) provide further support for the conclusions from those experimental studies. The calculated pre-edge feature for FeTPPPip_2_, arising from the nearly degenerate 1s to 3d_x2‑y2_ and 3d_z2_ transitions (Figure S27), is consistent with that expected for LS Fe­(II) centers in near-octahedral coordination environments. The shape of the calculated pre-edge feature for FeTPPPip is more complex, with an added shoulder on the higher-energy side of the peak due to the contribution of additional 1s to 3d transitions (Figure 6d and Figure S29). These observations are consistent with the expected d-orbital vacancies and energies afforded by HS Fe­(II) in square-pyramidal coordination environments, as previously reported.^45^ The linear combination of the calculated spectra of these 5- and 6-coordinate models in a 2:1 ratio yields a pre-edge feature (Figure 6d) that is in good agreement with the experimental spectrum of the piperidine-loaded MOF, further confirming the previously reported findings.

In summary, as the bulkiest nitrogenous base among the set of guest species evaluated in this study, piperidine axial ligation behavior in both MnCl-PCN-222-Pip and Fe-PCN-222-Pip deviates compared to that of their molecular porphyrin analogues. The pore size restrictions lead to the formation of unique coordination environments that cannot be achieved in the absence of the framework environments. Furthermore, the primary differences between the two MOFs lie in both their initial M-Cl distribution patterns and their reactivity toward piperidine guests. While the Fe porphyrin centers are reduced upon piperidine coordination, an analogous reduction of the Mn porphyrin centers is not observed under the same experimental conditions. The longer Mn-L bond length of the piperidine-coordinated Mn­(III) HS sites, combined with the initial distribution of Cl ligands in the framework, leads to a more crowded small-pore environment and, therefore, the lower ratio of penta- and hexacoordinated metal centers compared to the coordination environment in Fe-PCN-222-Pip.

### Bond Length Correlation with XAS Pre-Edge Feature Intensity

Experimental XANES data revealed that the pre-edge feature intensities of both the Fe and Mn complexes and the corresponding MOFs, depend on the bond length between the metal center and the ligands. Calculation results demonstrate a strong linear correlation between these bond lengths and the calculated oscillator strength for the 1s → 3d_x2‑y2_ transition (Figure 7). The intensity trend follows the expected changes associated with a reduced metal coordination environment symmetry and is consistent with previously reported results.^45,52^ For the Mn porphyrin species, the calculated oscillator strength for the 1s → 3d_x2‑y2_ transition increases with the M–L bond length and decreases with the M–Cl bond distance for hexacoordinated species. For the Mn porphyrin species, the Mn–L bond length increases in the following order: MnTPPIm_2_^2+^ < MnTPPPip_2_^2+^ < MnClTPPPy < MnClTPPDABCO, MnClTPPIm < MnClTPPPip. A similar trend is observed for the Fe porphyrin series, yet with more complex behavior (Figure S30), dictated by the oxidation and spin states of the Fe sites, as well as their participation in π-backbonding, both of which influence the metal center electron density and radius.^45,52^ Nevertheless, the combination of XAS and TD-DFT calculation results provides valuable insight into the symmetry of the metal center and M–L bond length, and can be used to assess the bond strengths and lengths in complex systems such as MOFs.

**Figure 7:**
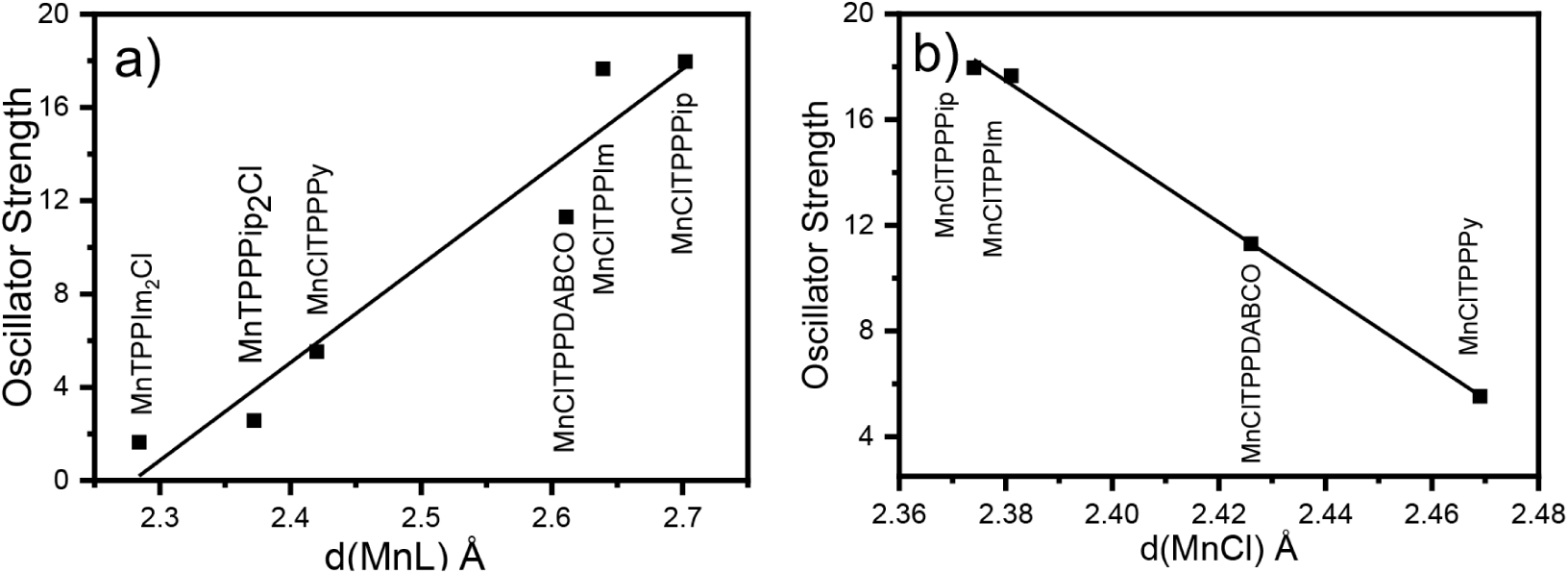
Correlation between (a) Mn–L and (b) Mn–Cl calculated bond lengths and calculated oscillator strength for 1s → 3d_x2‑y2_ in MnClTPPL and MnTPPL_2_ model complexes, where L is a nitrogenous base axial ligand.

## Conclusion

This study demonstrates how the structural differences between Mn-PCN-222 and Fe-PCN-222 ultimately affect their host–guest interactions across a range of axially binding guest molecules in ways that sometimes deviate from expected behavior based on their molecular porphyrin analogues. Differences in the initial distribution of small and large pore-facing chloride ligands, as well as porphyrin ring planarity and M–L bond length—dictated by the metal center oxidation and spin state changes—lead to significantly different axial ligation outcomes. For the manganese porphyrin MOF, the metal center remains high-spin Mn­(III), and the distribution between penta- and hexa-coordinated centers tracks with the initial Cl^–^ ligand orientation distribution in most cases. For the Fe porphyrin MOF, however, we observe substantially different behavior, since the guest size and reactivity of the metal center play larger roles in dictating the coordination pattern. This study showcases the use of X-ray absorption spectroscopy with TD-DFT calculation support to establish local geometry variations in complex MOF–guest systems and lays a foundation for the application of this combination of methods in studying other host–guest interactions in these types of frameworks, such as those relevant to catalytic processes.

## Supplementary Material



## Notes

Powder and single-crystal X-ray diffraction methods and results, UV–vis absorption and diffuse reflectance spectra, additional XANES and linear combination fit data, results and details of IAD analysis of XES spectra, additional TD-DFT calculation results, and additional information on experimental synthesis and characterization methods (PDF)
