# Supplementary material for: Framework-Controlled Axial Coordination of Guest Molecules in Metalloporphyrin-Based MOFs
Source: Inorg Chem. 2026 Jul 16;65(29):16663–74. doi: 10.1021/acs.inorgchem.6c00778 (PMC13418171; doi:10.1021/acs.inorgchem.6c00778)
Supplement: Supplementary file 1 [file ic6c00778_si_001.pdf]

Framework-controlled axial coordination of guest molecules in metalloporphyrin-based MOFs

Alison Arissa,[a] Nicole Lahanas,[a] Maaz Afzal,[a] Roger Lalancette,[a] Pavel Kucheryavy,[a] Jenny V. Lockard[a]**

[a] Department of Chemistry, Rutgers University, Newark, NJ 07102 USA

* pvk10@newark.rutgers.edu, * jlockard@newark.rutgers.edu

Supporting Information

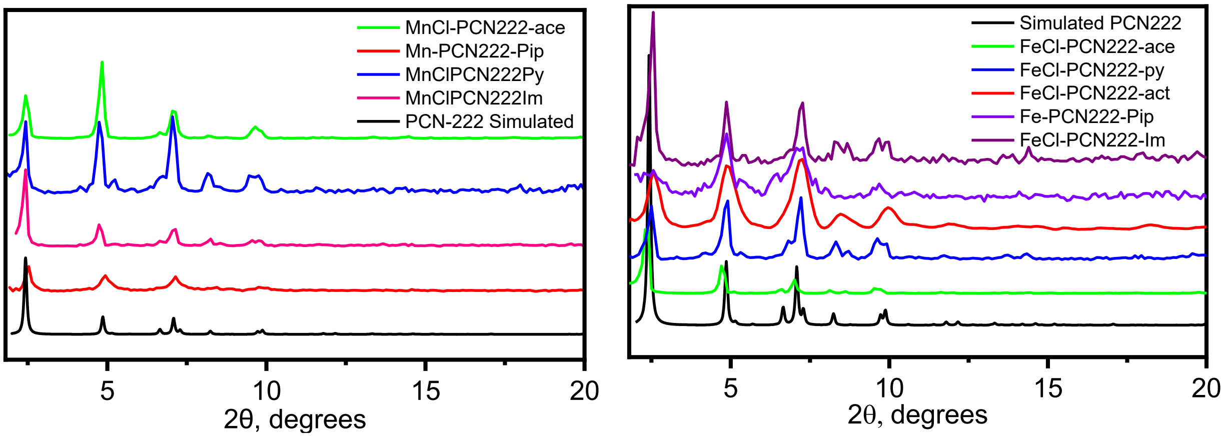

Figure S1. Powder XRD patterns for MnCl-PCN222 and FeCl-PCN222 [1] MOFs.

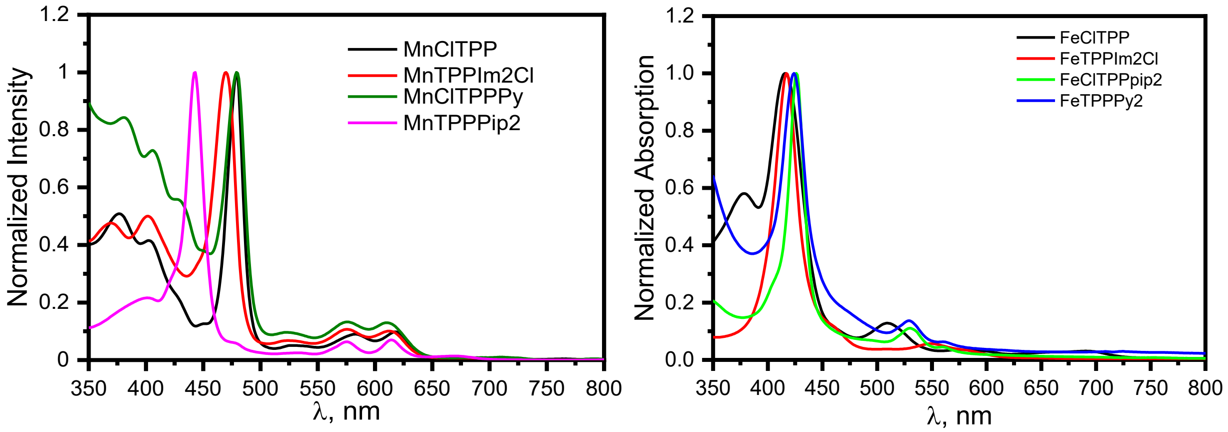

Figure S2. UV-Vis absorption spectra of Mn-Porphyrin and Fe-Porphyrin complexes in dichloromethane solvent with different axial ligands.

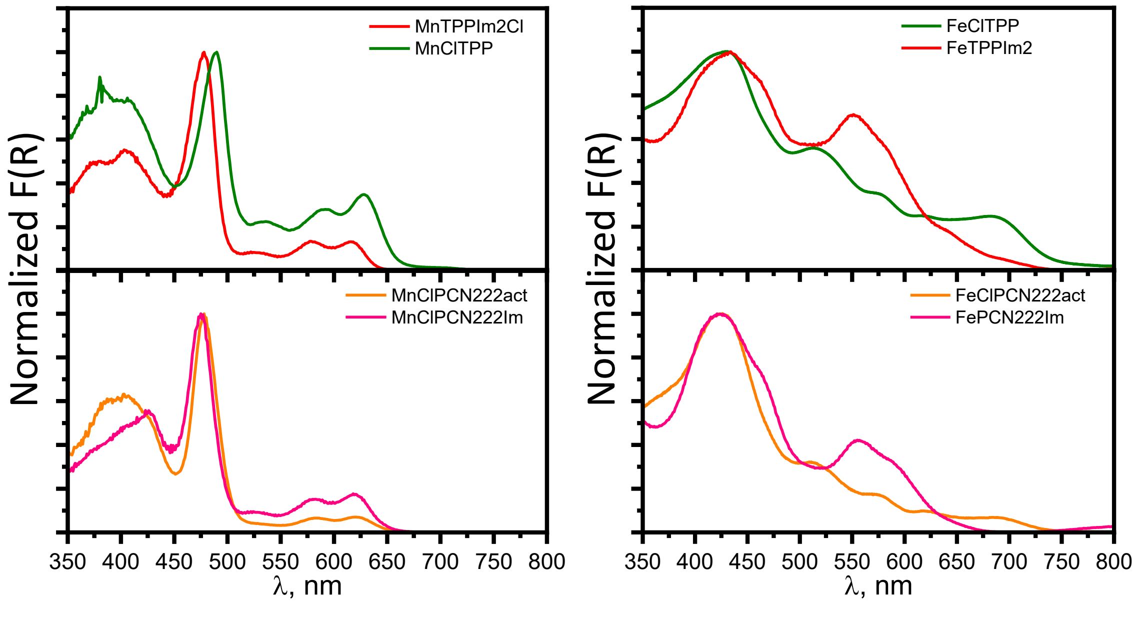

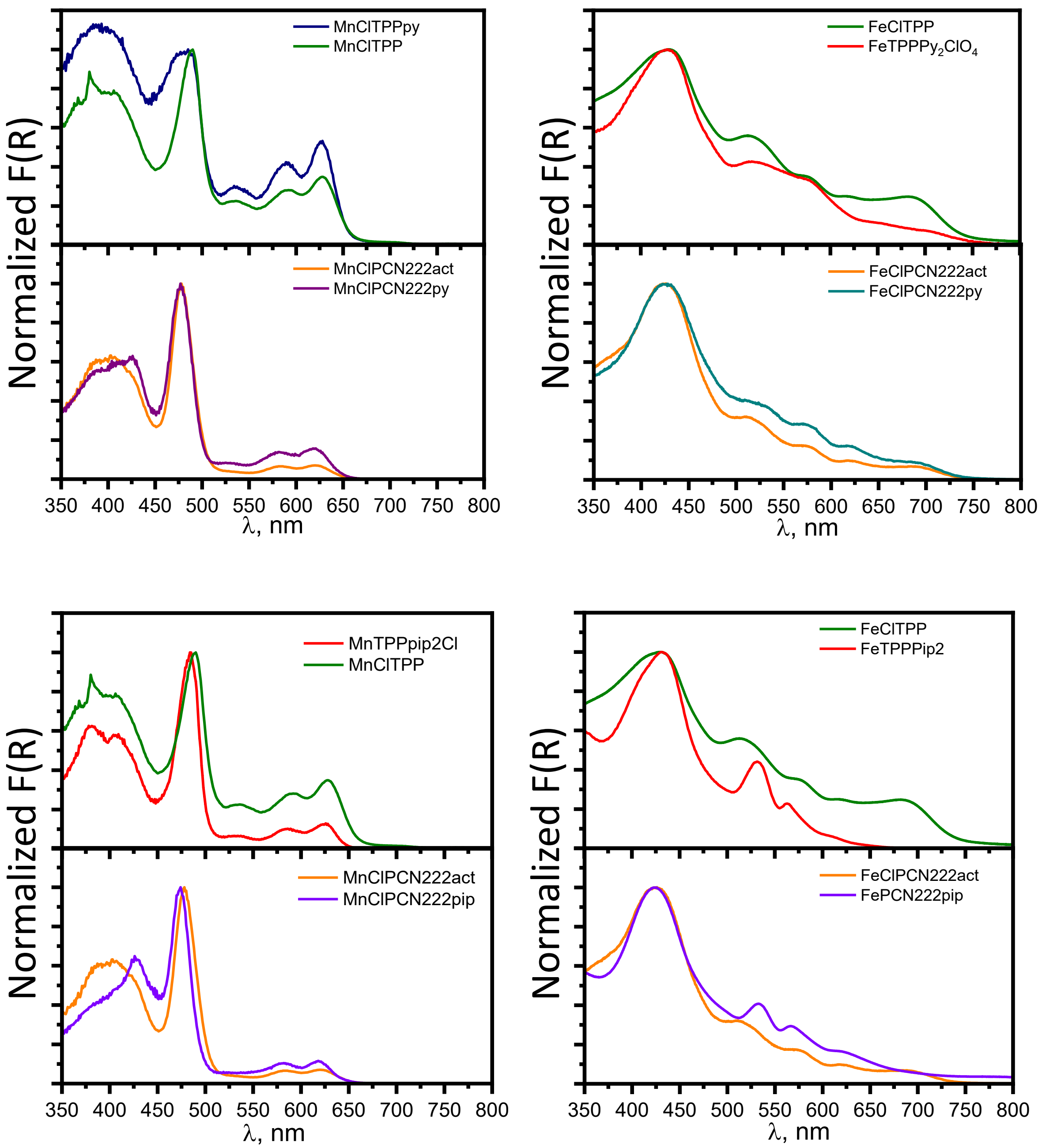

Figure S3. Diffuse reflectance spectra of PCN222 under different guest environments and corresponding tetraphenyl porphyrin (TPP) reference complexes

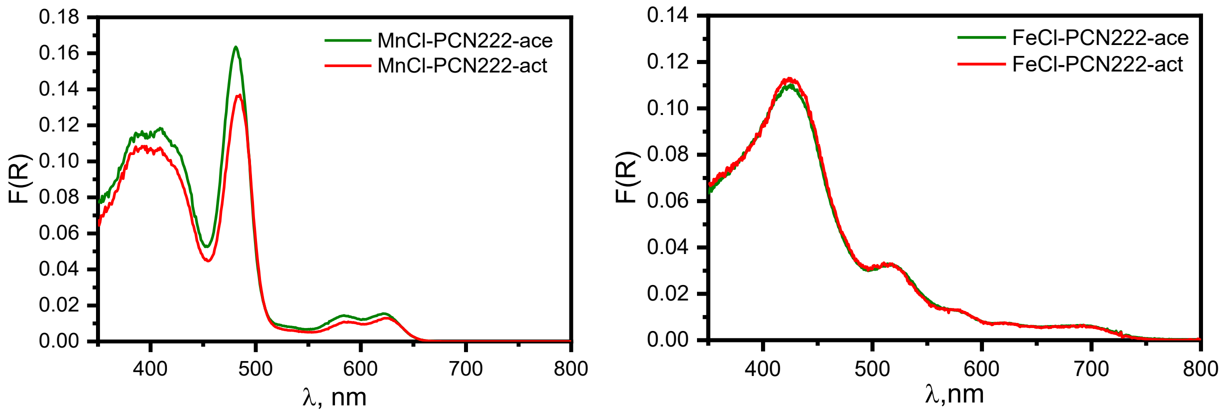

Figure S4. In situ diffuse reflectance spectra of acetone treated and activated PCN222.

Table S1. Diffuse reflectance band maxima for Mn-porphyrin reference complexes

	MnClTPP	$MnTPPIm_2Cl$	MnClTPPPy	$MnTPPPip_2Cl$
λ, nm	490<br>534<br>590<br>628	478<br>525<br>578<br>616	485<br>534<br>590<br>627	484<br>532<br>586<br>625

Table S2. Diffuse reflectance band maxima Mn-PCN222 with different guest environments.

	MnCl-PCN222-act	MnCl-PCN222-ace	MnCl-PCN222-Im	MnCl-PCN222-Py	MnCl-PCN222-Pip
λ, nm	478<br>528<br>584<br>621	482<br>526<br>583<br>622	475<br>524<br>582<br>620	477<br>527<br>583<br>620	474<br>522<br>581<br>618

Table S3. Diffuse reflectance band maxima for Fe-porphyrin reference complexes

	FeClTPP	$FeTPPIm_2Cl$	$FeTPPPy_2ClO_4$	$FeTPPPip_2$
λ, nm	428<br>513<br>565<br>618<br>682	434<br>551<br>582	430<br>516<br>574	430<br>531<br>562

Table S4. Diffuse reflectance band maxima for Fe-porphyrin reference complexes

	FeCl-PCN222-act	FeCl-PCN222-ace	FeCl-PCN222-Im	FeCl-PCN222-Py	Fe-PCN222-Pip
λ, nm	428<br>510<br>571<br>619<br>686	428<br>510<br>571<br>619<br>689	424<br>555<br>588	425<br>519<br>572<br>618<br>690	423<br>532<br>566<br>610

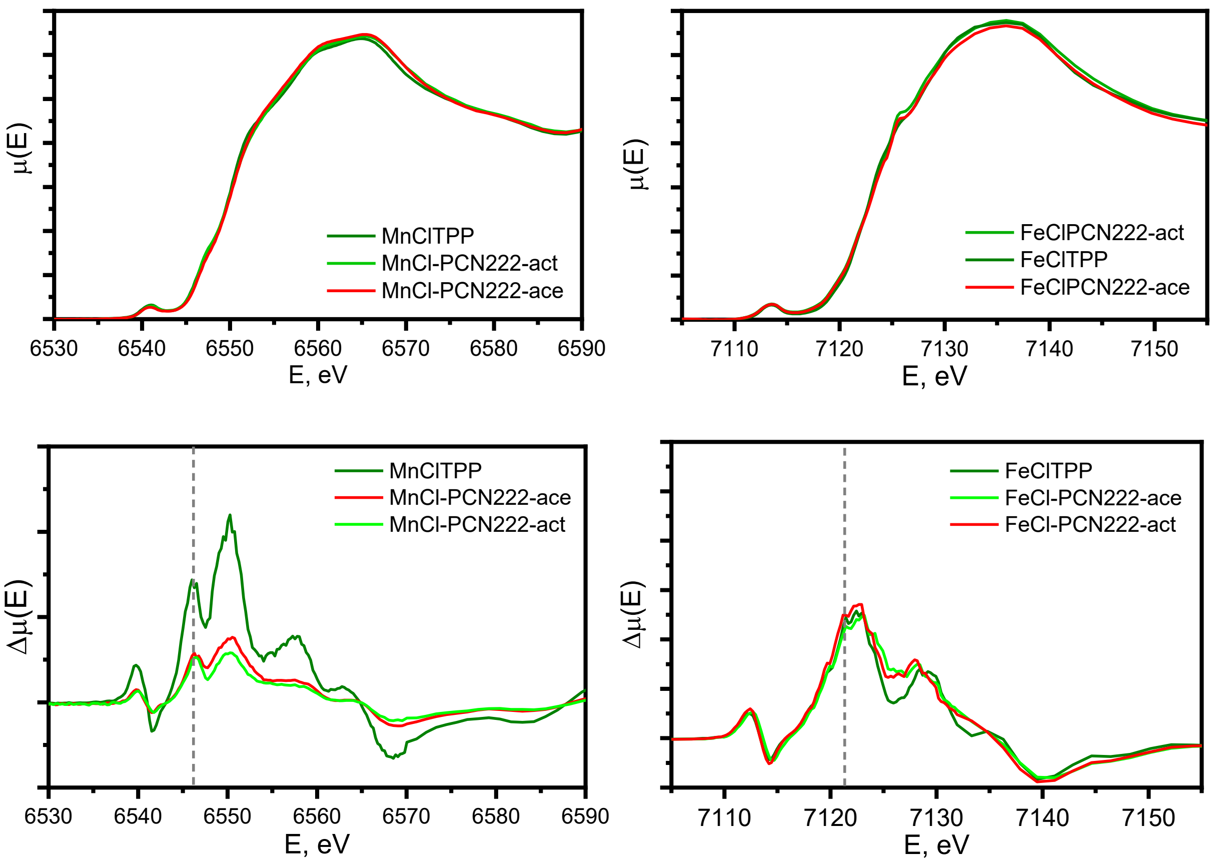

Figure S5. XANES (top) and associated derivative spectra (bottom) for Mn (left) and Fe[1] (right) porphyrin reference complexes and corresponding MOFs with acetone or activated pore environment.

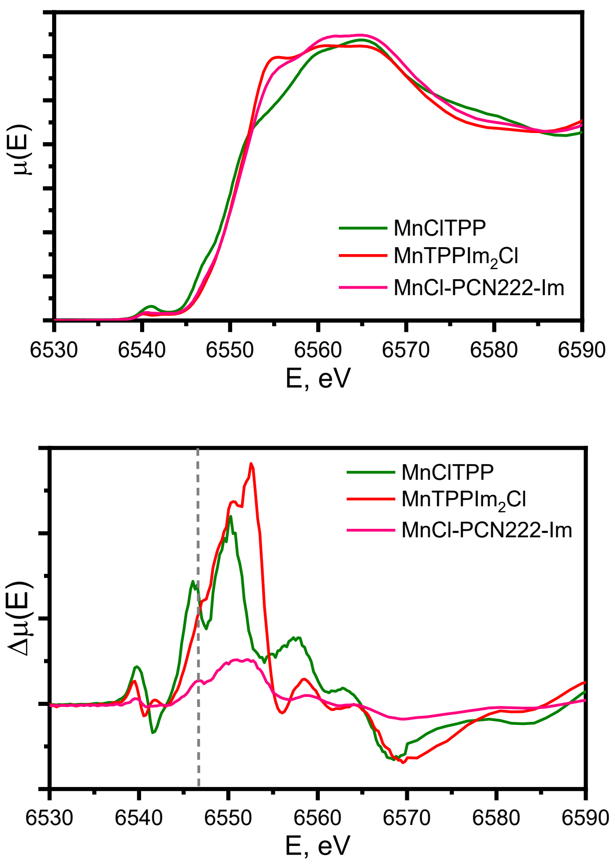

Figure S6. XANES (top) and associated derivative spectra (bottom) for Mn (left) and Fe[1] (right) porphyrin reference complexes and corresponding MOFs with imidazole guest environment.

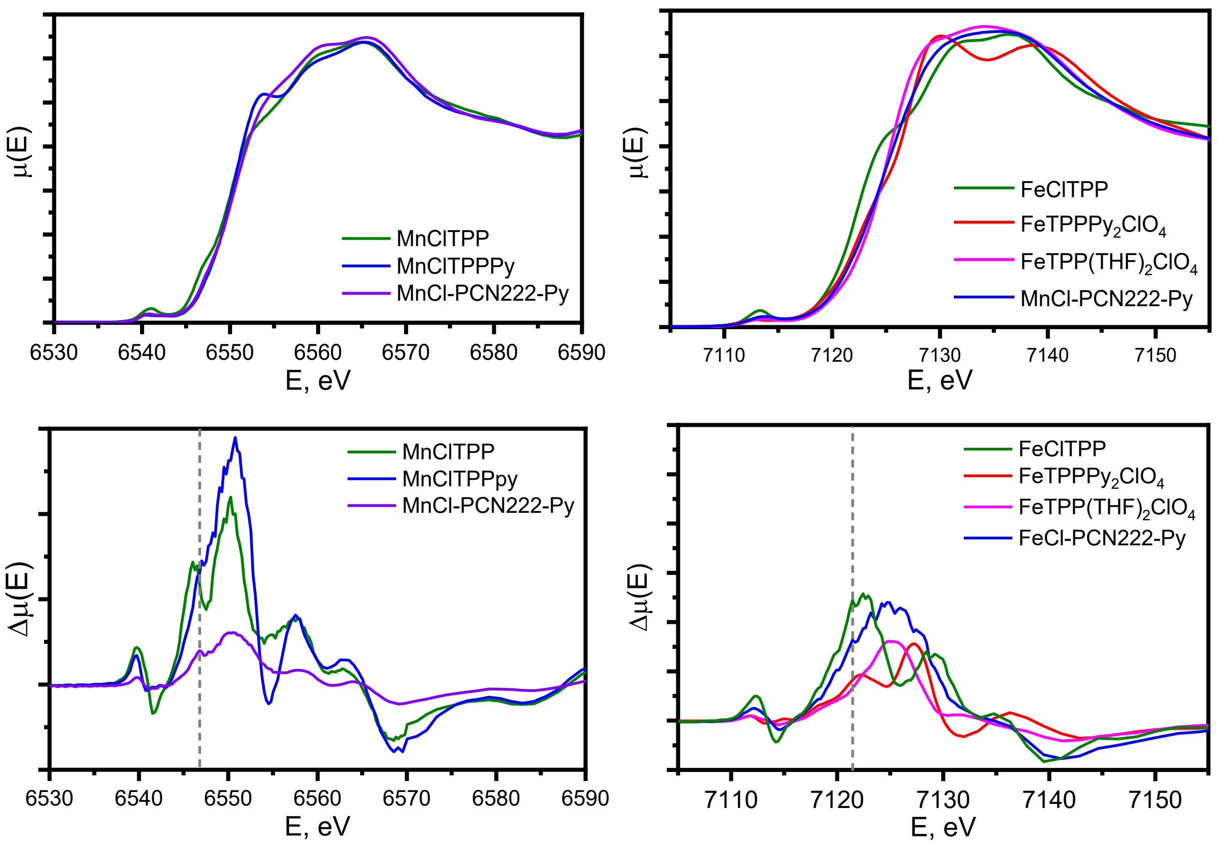

Figure S7. XANES (top) and associated derivative spectra (bottom) for Mn (left) and Fe (right) porphyrin reference complexes and corresponding MOFs with pyridine guest environment.

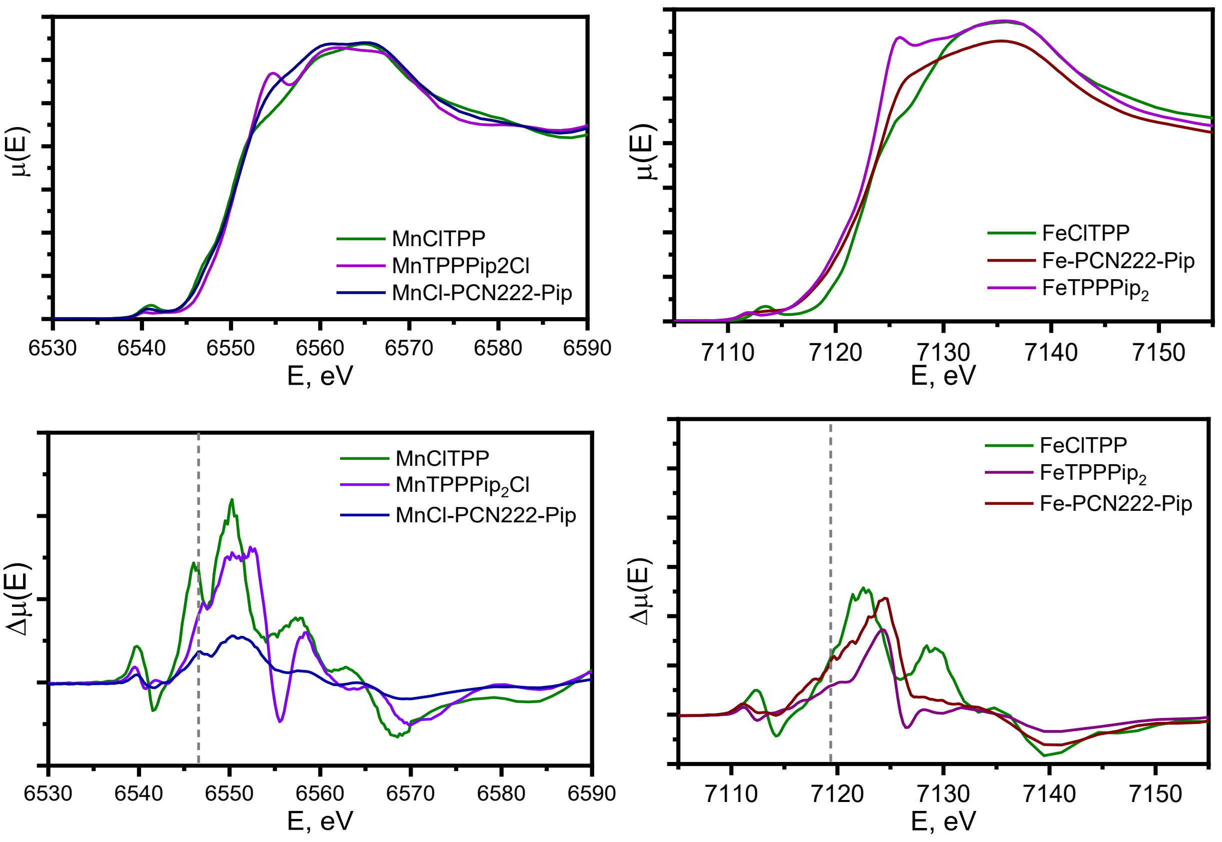

Figure S8. XANES (top) and associated derivative spectra (bottom) for Mn and Fe[1] porphyrin reference complexes and corresponding MOFs with piperidine guest environment.

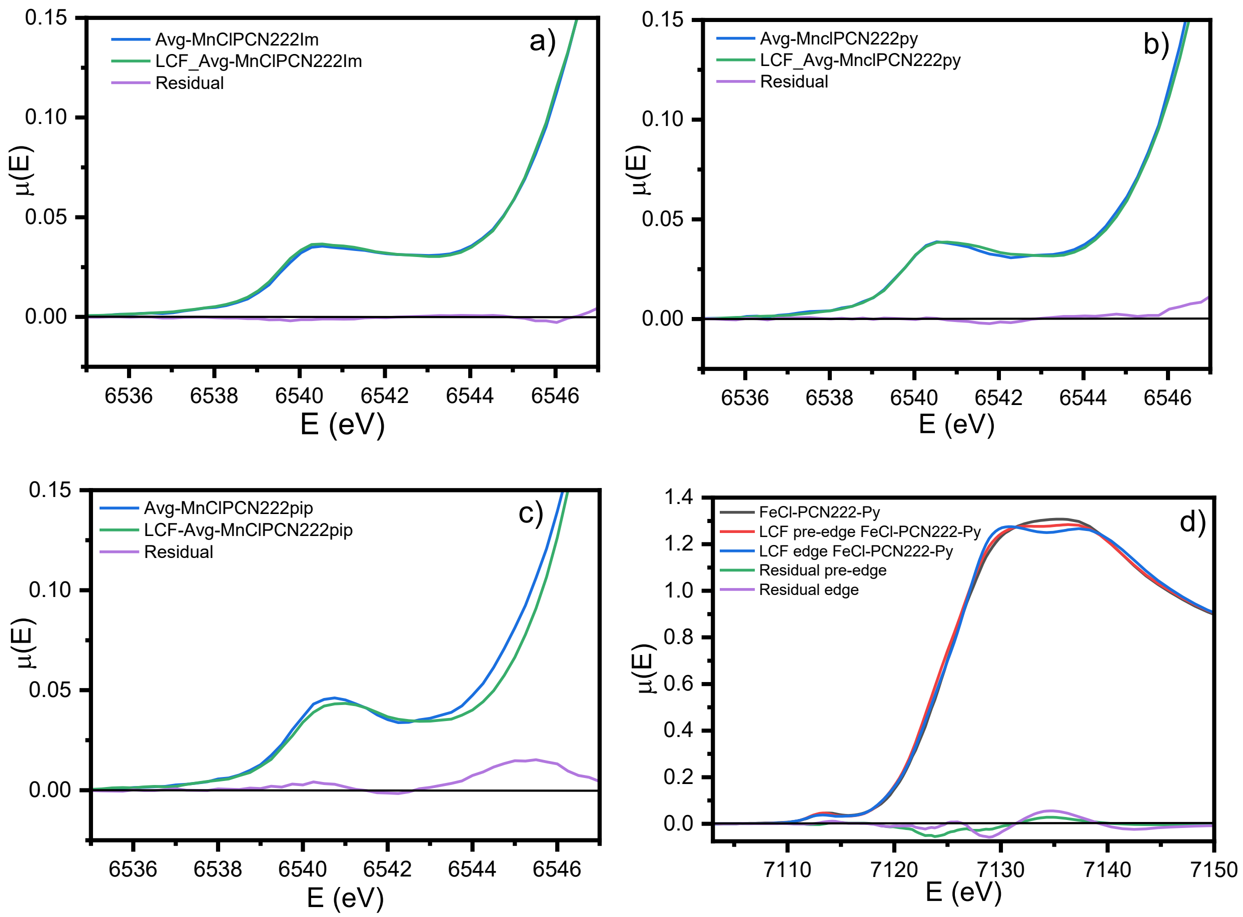

Figure S9. Experimental data linear combination fits, and residuals for a) MnCl-PCN222-Im, b) MnCl-PCN222-Py, c) MnCl-PCN222-Pip, and d) FeCl-PCN222-Py.

Table S5. Fit Results for MnCl-PCN222-L MOFs

	MnCl-PCN222-Im	MnCl-PCN222-Py	MnCl-PCN222-Pip
Standards	MnCl-PCN222-act, $MnTPPIm_2Cl$	MnCl-PCN222-act, MnClTPPPy	MnCl-PCN222-act, $MnTPPPip_2Cl$
Ratio	0.267:0.733 (1:2.74)	0.217:0.784 (1:3.61)	0.553:0.447 (1:0.81)
R-factor	3×10^{-4}	4×10^{-7}	2×10^{-6}

Table S6. Fit results for FeCl-PCN222-Py with focus on pre-edge (7108.28 – 7118.28 eV) and on edge (7108.28 – 7128.28 eV)

Region	7108.28 – 7118.28 eV	7108.28 – 7128.28 eV
Standards	$FeTPPPy_2ClO_4$, $FeTPP(THF)_2ClO_4$, FeClTPP	$FeTPPPy_2ClO_4$, $FeTPP(THF)_2ClO_4$, FeClTPP
Ratio	0.237:0.446:0.115 (0.75:1.41:1)	0.497:0.338:0.115 (4.32:3.37:1)
R-factor	2.96×10^{-2}	1.47×10^{-3}

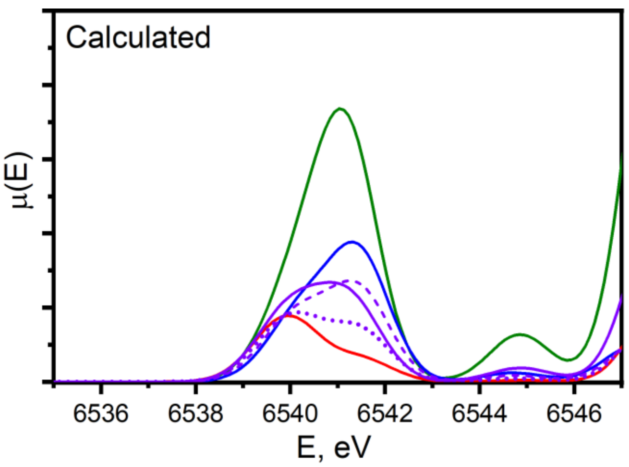

Figure S10. Calculated XAS for MnClTPP (green), $MnTPPIm_2Cl$ (red), MnClTPPIm (blue) overlaid with the following linear combinations of calculated spectra: 26.7% MnClTPP and 73.3% $MnTPPIm_2Cl$ (solid purple), 73% MnClTPPIm and 27% $MnTPPIm_2Cl$ (dashed purple) and 65% MnClTPPIm and 35% $MnTPPIm_2Cl$ (dotted purple).

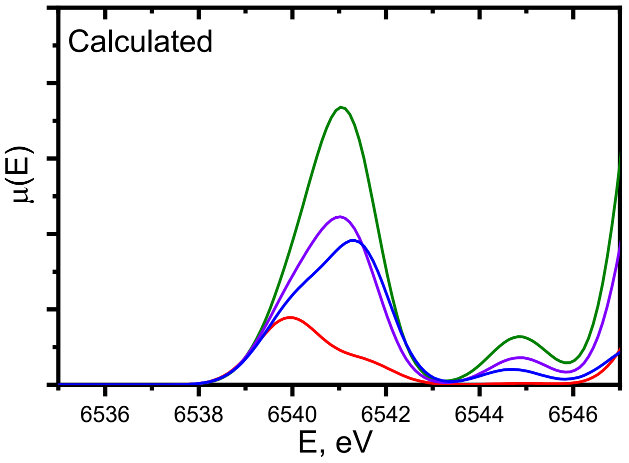

Figure S11. Calculated XAS for MnClTPP (green), $MnTPPPip_2Cl$ (red), MnClTPPPip (blue), linear combination of 55.3% MnClTPP and 44.7% $MnTPPPip_2Cl$ calculated spectra (purple)

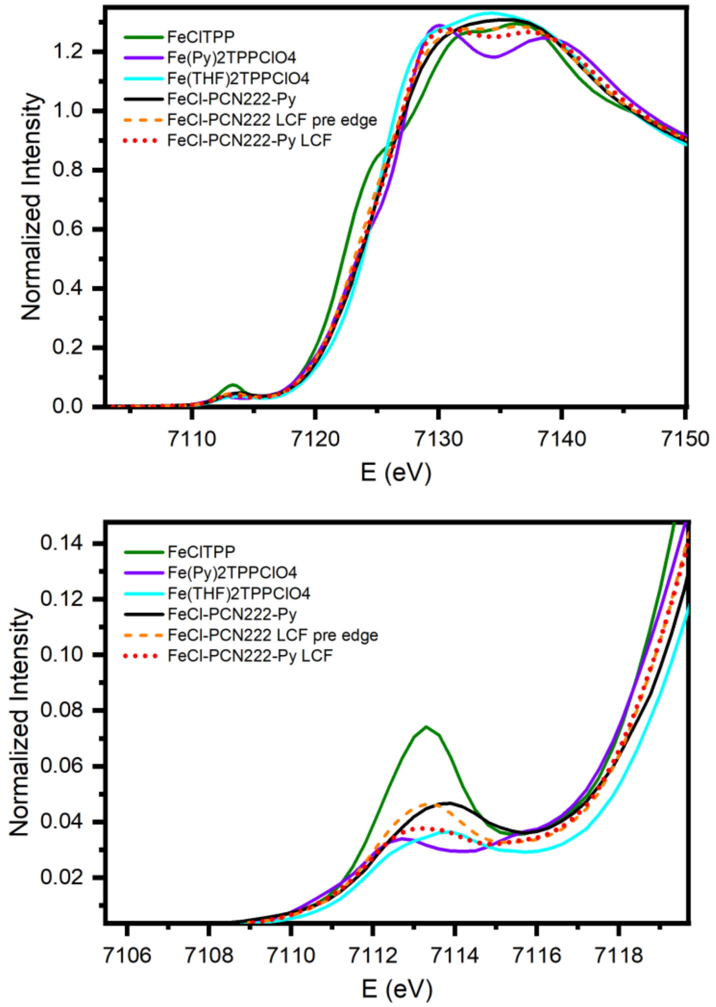

Figure S12. XANES Linear combination fits (LCF) for FeCl-PCN222-Py using reference complex experimental spectra.

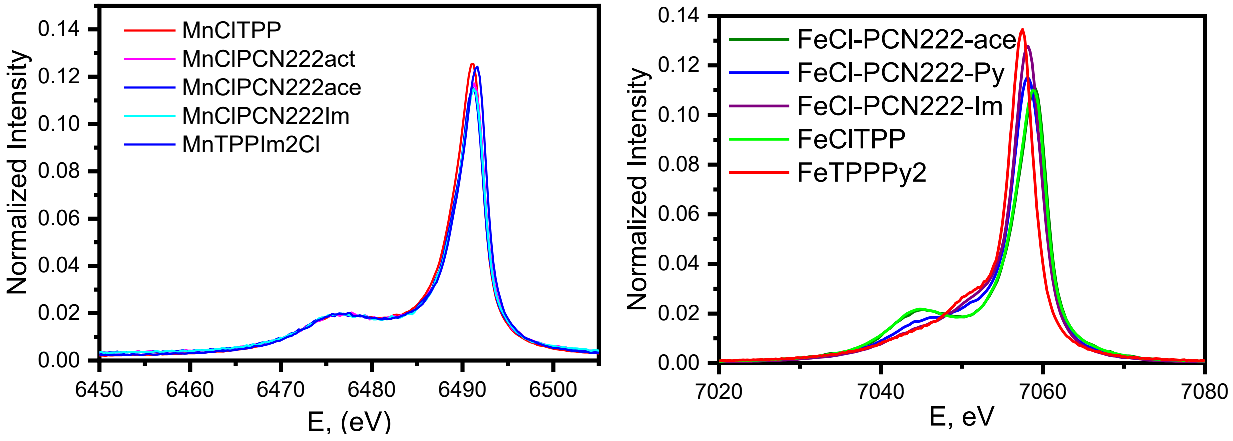

Figure S13. XES spectra for Mn reference complexes and MOFs (left), and FeCl-PCN222 MOFs and reference complexes.[1]

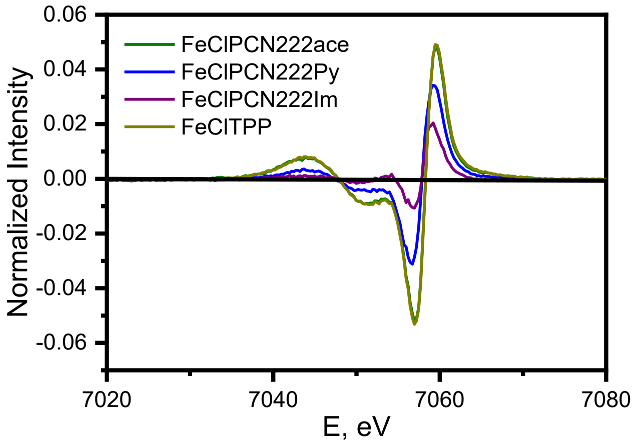

Figure S14. Difference spectra for IAD analysis obtained between spectra of *HS(E)* = FeClTPP, MS(E) = FeCl-PCN222-ace, FeCl-PCN222-Py, or FeCl-PCN222-Im and that of LS(E) = FeTPPpy2 LS reference complex

The following equations were used to calculate the average spin values for compounds with an unknown spin state:[2]

$$\Delta S_{ML} = \frac{IAD_{ML} \times \Delta S_{HL}}{IAD_{HL}}$$

$IAD_{HL} = \int |[HS(E) - LS(E)]| dE$

$IAD_{ML} = \int |[MS(E) - LS(E)]| dE$

IAD_{HL} – absolute area of the high spin – low spin XES difference spectrum

IAD_{ML} – absolute area of the unknown spin – low spin XES difference spectrum

ΔS_{HL} – difference between spin state values for high and low spin reference compounds.

ΔS_{ML} - difference between spin state values of unknown compound and the low spin reference.

Table S7. IAD analysis results for FeCl-PCN222-X MOFs

	IAD	S	Expected S	Ratio
FeClPCN222ace[1]	0.36	2.45	2.5	98% HS and 2% LS
FeClPCN222py	0.23	1.55	2.5 and 0.5	53% HS and 47%LS
FeClPCN222Im[1]	0.12	0.81	0.5	15% HS and 85% LS

Table S8. XAS and XES data for the reference complexes and MOFs

			XAS		XES		attribution
	Ox	S_{eff}	Pre-edge, eV	Edge, eV	$K\beta_{1,3}$, eV	$K\beta'$, eV	
MnClTPP	+3	2	6541.0	6546.01	6490.95	6476.95	-[a]
MnCl-PCN222-ace	+3	2	6541.0	6546.25	6491.19	6476.44	-[a]
MnCl-PCN222-act	+3	2	6541.0	6546.25	6491.28	6476.28	-[a]
MnTPPIm2Cl	+3	2	6540.2, 6542.75	6547.2	6491.64	6476.39	-[a]
MnCl-PCN222-Im	+3	2	6540.5, 6542.75	6546.75	6491.2	6476.45	-[a]
MnClTPPPy	+3	2	6540.5, 6542.75	6547.0	-	-	-[a]
MnCl-PCN222-Py	+3	2	6540.75	6546.75	-	-	-[a]
$MnTPPPip_2Cl$	+3	2	6540.25, 6542.75	6547.0	-	-	-[a]
MnCl-PCN222-Pip	+3	2	6540.75	6546.75, 6547.5	-	-	-[a]
MnClTPPdabco	+3	2	6540.75	6547.25	-	-	-[a]
MnCl-PCN222-dabco	+3	2	6540.5	6547.25	-	-	-[a]
FeClTPP	+3	2.5	7113.3	7122.3	7058.7	7044.7	ref. 1
FeCl-PCN222-ace	+3	2.5	7113.6	7122.5	7058.7	7044.7	ref. 1
FeCl-PCN222-act	+3	2.5	7113.3	7122.5	7058.9	7044.7	ref. 1
$FeTPPIm_2Cl$	+3	0.5	7111.2, 7112.9	7122.0	7057.7	7045.2	ref. 1
FeCl-PCN222-Im	+3	0.5	7111.5, 7112.9	7122.0	7057.7	7045.2	ref. 1
$FeTPPPy_2ClO_4$	+3	0.5	7110.9, 7112.6	7122.3	-	-	-[a]
$FeTPP(THF)_2ClO_4$	+3	2.5	7112.8, 7113.9	7124.5	-	-	-[a]
FeCl-PCN222-Py	+3	1.5	7113.6	7121.5	7058.4	7044.7	-[a]
$FeTPPPip_2$	+2	0	7112.0	7120.5	7057.3	7044.0	ref. 1
Fe-PCN222-Pip	+2	1.33	7112.1, 7113.2	7120.5	7057.6	7044.3	ref. 1

[a]this work

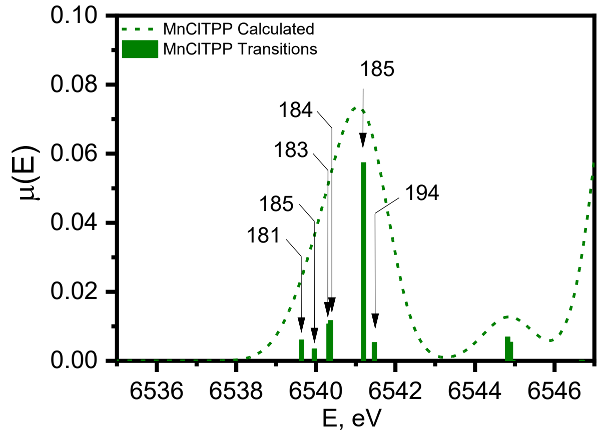

Figure S15. Calculated pre-edge feature and underlying transitions for MnClTPP

Table S9. Transitions, orbitals, oscillator strength for MnClTPP

Transition	Orbital	Major AO contributions	Oscillator Strength	Visual rendering of the MOs
1	181β	d_{xy}	3.35	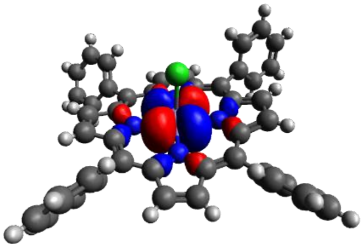
2	185α	d_{z2}	1.92	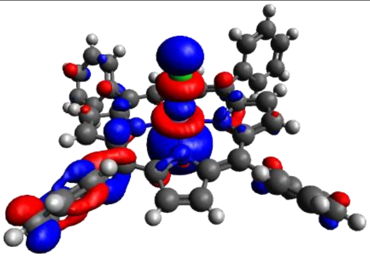
3	183β	d_{xz}	5.88	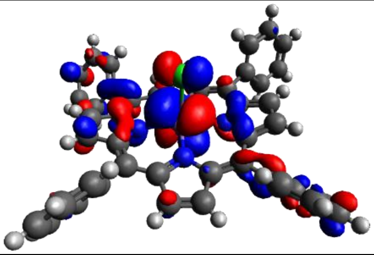

4	184β	d_{yz}	6.42	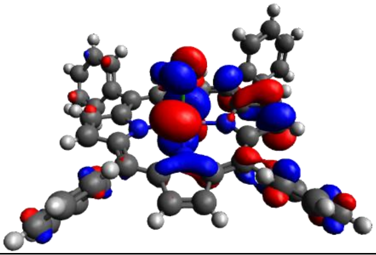
5	185β	d_{z2}	31.59	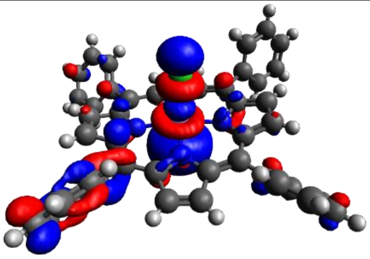
6	194β	d_{x2-y2}	2.93	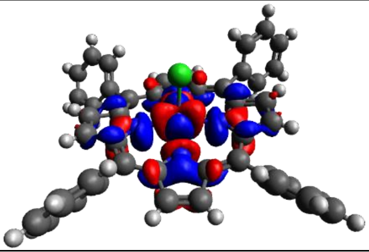

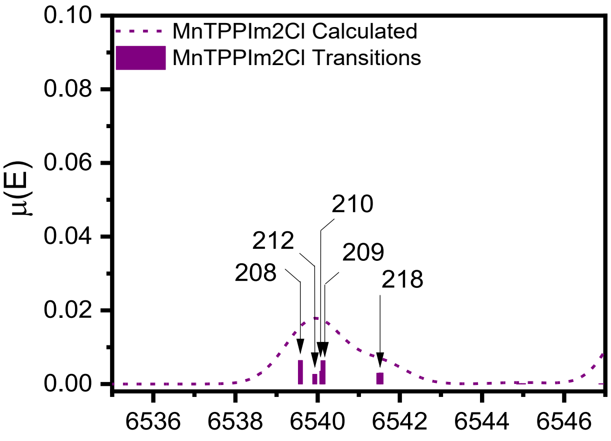

Figure S16. Calculated pre-edge feature and underlying transitions for $MnTPPIm_2Cl$

Table S10. Transitions, orbitals, oscillator strength for $MnTPPIm_2Cl$

Transition	Orbital	Major AO contributions	Oscillator Strength	Visual rendering of the MOs
1	208β	d_{xy}	3.74	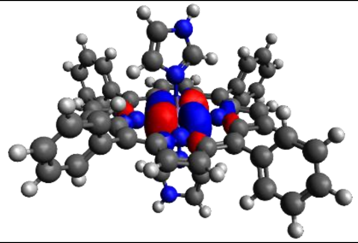
2	212α		2.13	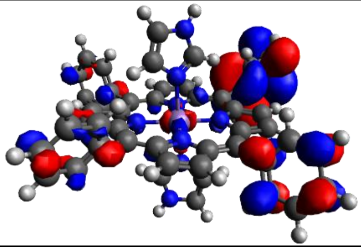
3	210β	d_{xz}	3.77	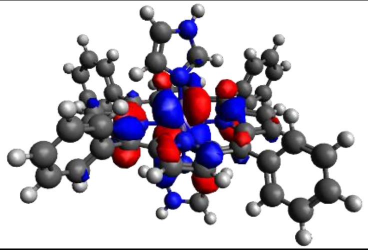
4	209β	d_{yz}	3.75	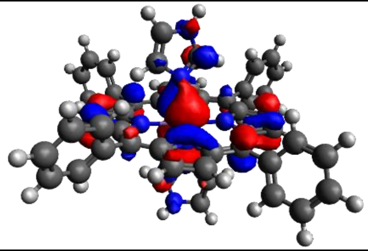

5	218β	d_{x2-y2}	2.5	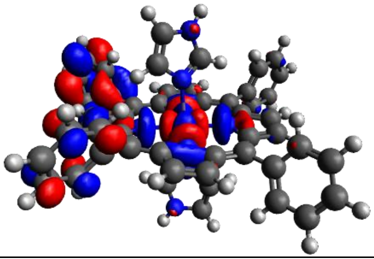
6	221β	d_{z2}	2.31	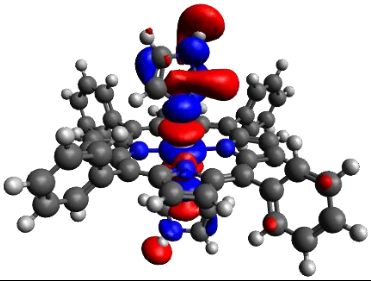

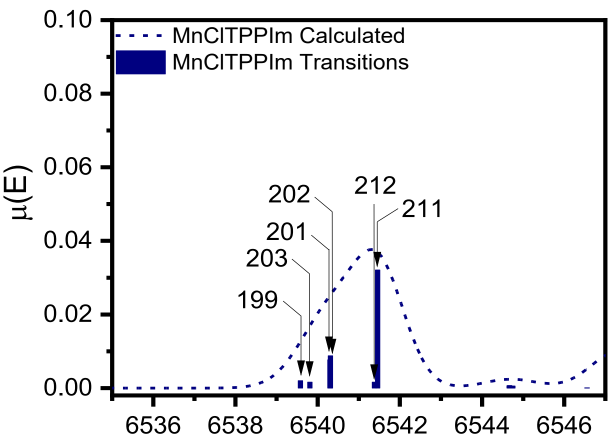

Figure S17. Calculated pre-edge feature and underlying transitions for MnClTPPIm

Table S11. Transitions, orbitals, oscillator strength for MnClTPPIm

Transition	Orbital	Major AO contributions	Oscillator Strength	Visual rendering of the MOs
1	199β	d_{xy}	3.55	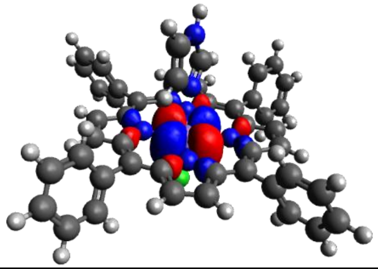
2	203α	d_{xz}	2.24	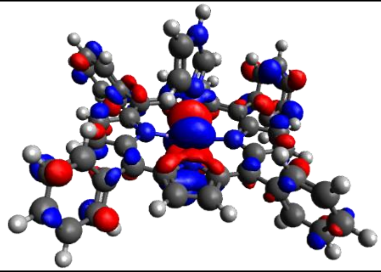
3	201β	d_{yz}	10.47	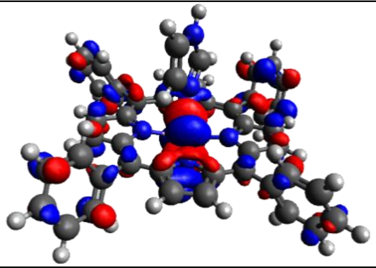

4	202β	$d_{xz} + d_{yz}$	11.96	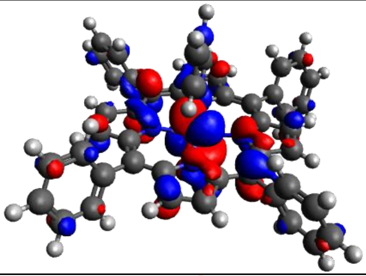
5	212β	d_{x2-y2}	2.58	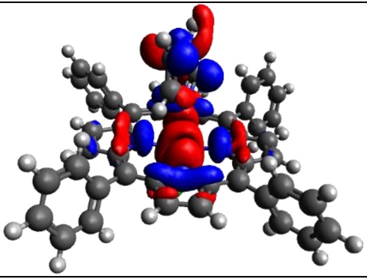
6	211β	d_{z2}	18.59	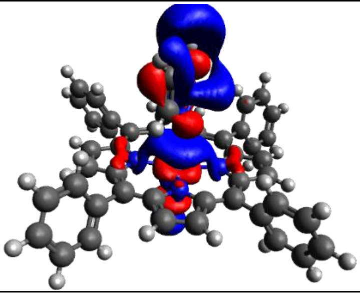

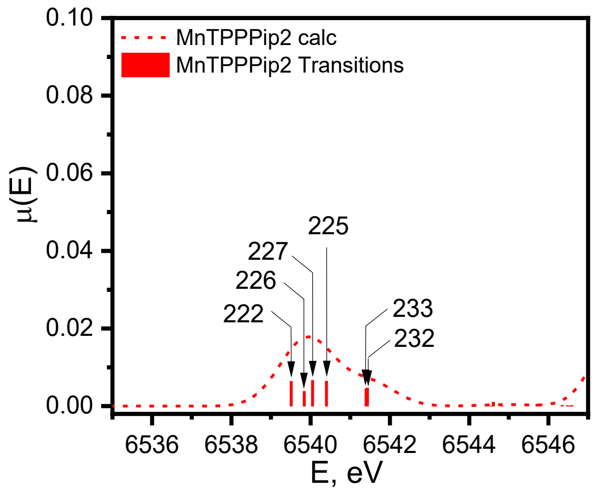

Figure S18. Calculated pre-edge feature and underlying transitions for $MnTPPPip_2Cl$

Table S12. Transitions, orbitals, oscillator strength for $MnTPPPip_2Cl$

Transition	Orbital	Major AO contributions	Oscillator Strength	Visual rendering of the MOs
1	222β	d_{xy}	3.51	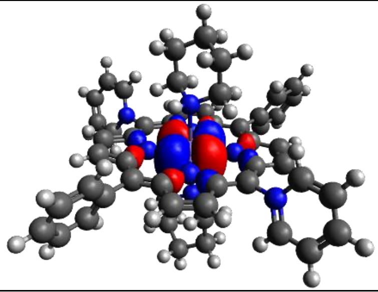
2	226α		2.08	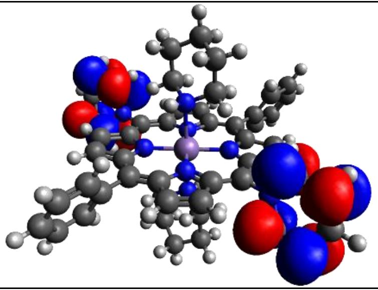
3	227β	$d_{xz} + d_{yz}$	3.64	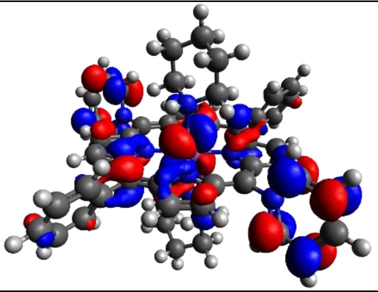

4	225β	$d_{xz} + d_{yz}$	3.52	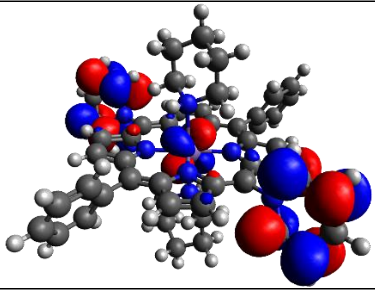
5	233β	d_{x2-y2}	2.44	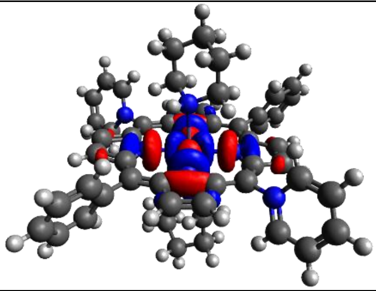
6	232β	d_{z2}	2.57	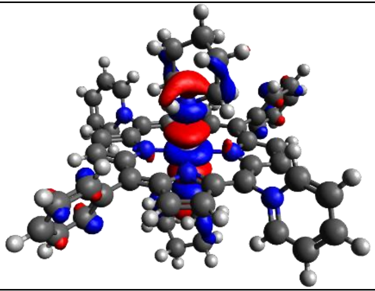

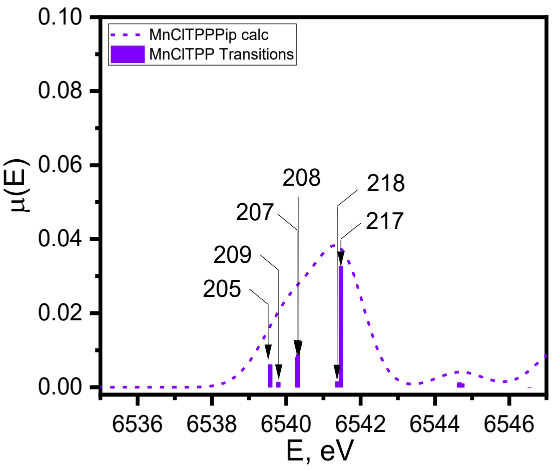

Figure S19. Calculated pre-edge feature and underlying transitions for MnClTPPPip

Table S13. Transitions, orbitals, oscillator strength for MnClTPPPip

Transition	Orbital	Major AO contributions	Oscillator Strength	Visual rendering of the MOs
1	205β	d_{xy}	3.55	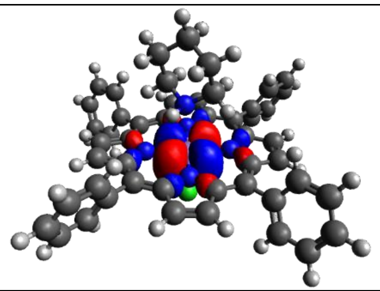
2	209α		2.24	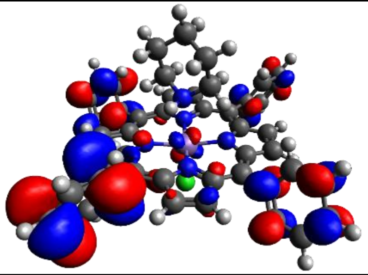
3	207β	$d_{xz} + d_{yz}$	10.47	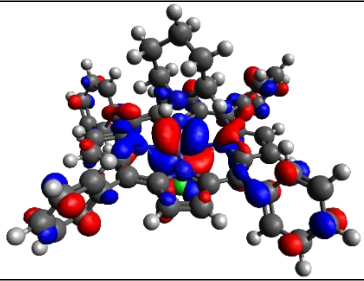

4	208β	$d_{xz} + d_{yz}$	11.96	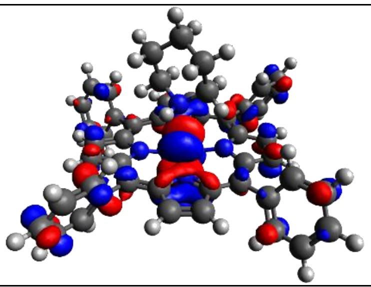
5	218β	d_{x2-y2}	2.58	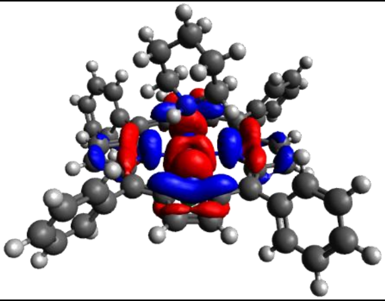
6	217β	d_{z2}	18.59	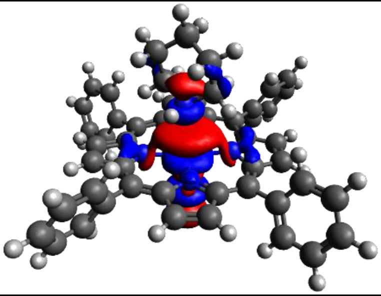

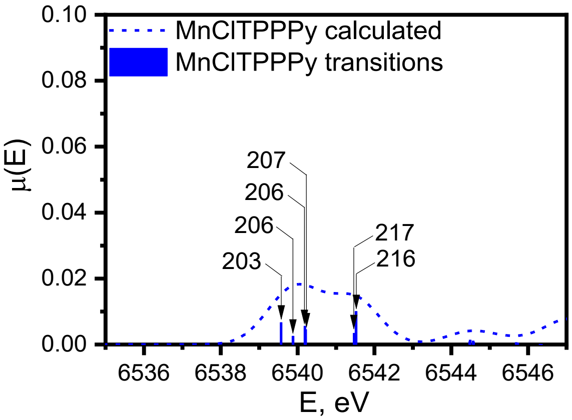

Figure S20. Calculated pre-edge feature and underlying transitions for MnClTPPPy

Table S14. Transitions, orbitals, oscillator strength for MnClTPPPy

Transition	Orbital	Major AO contributions	Oscillator Strength	Visual rendering of the MOs
1	203β	d_{xy}	3.65	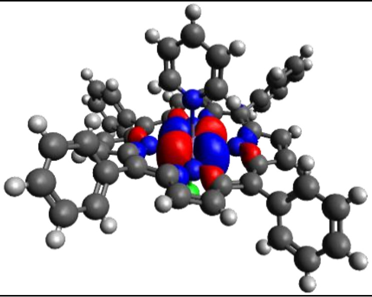
2	206α	$d_{xz} + d_{yz}$	1.38	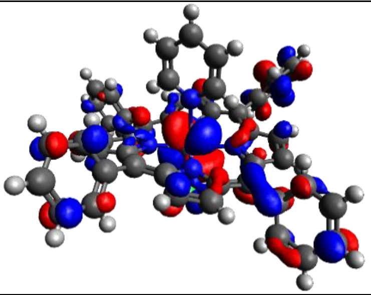
3	206β	$d_{xz} + d_{yz}$	3.08	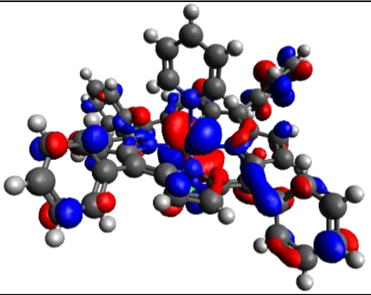

4	207β	$d_{xz} + d_{yz}$	2.51	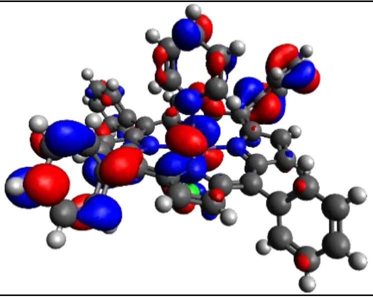
5	217β	d_{x2-y2}	1.89	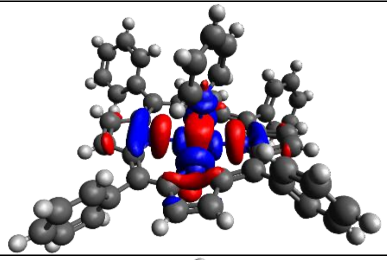
6	216β	d_{z2}	5.53	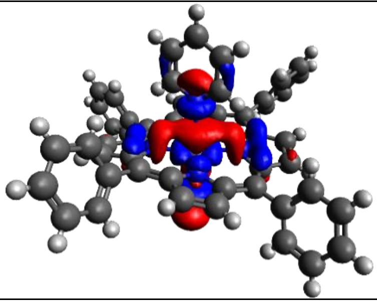

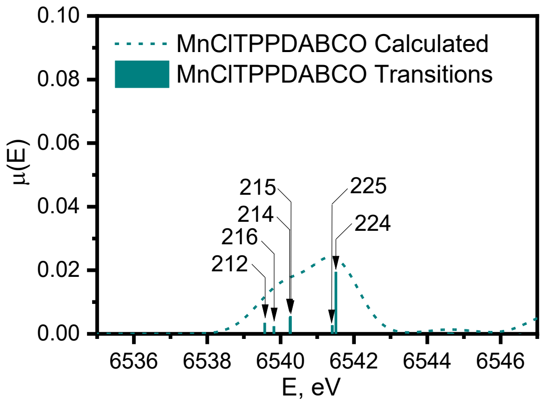

Figure S21. Calculated pre-edge feature and underlying transitions for MnClTPPDABCO

Table S15. Transitions, orbitals, oscillator strength for MnClTPPDABCO

Transition	Orbital	Major AO contributions	Oscillator Strength	Visual rendering of the MOs
1	212β	d_{xy}	3.57	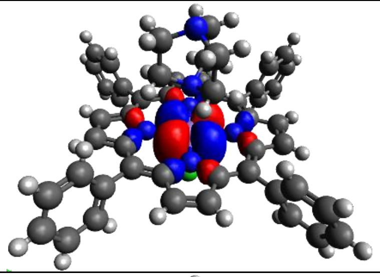
2	216α		2.23	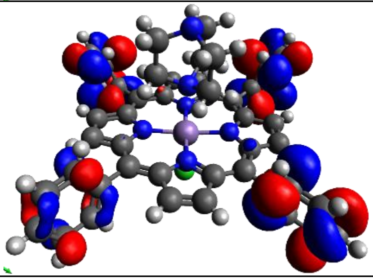
3	214β	d_{xz}	6.98	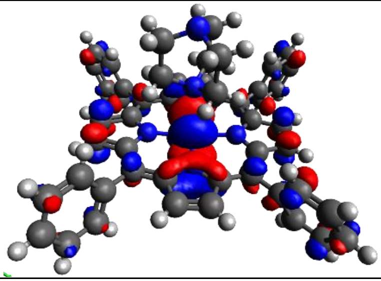

4	215β	d_{yz}	7.62	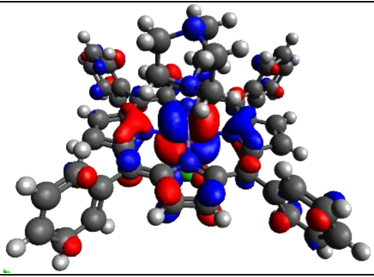
5	225β	d_{x2-dy2}	2.56	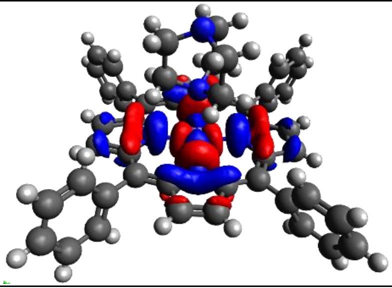
6	224β	d_{z2}	11.31	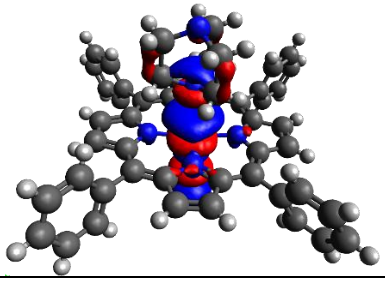

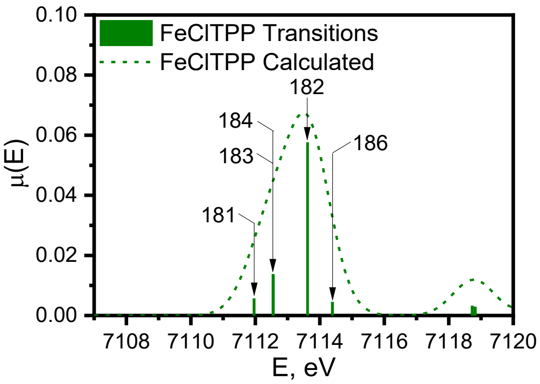

Figure S22. Calculated pre-edge feature and underlying transitions for FeClTPP

Table S16. Transitions, orbitals, oscillator strength for FeClTPP

Transition	Orbital	Major AO contributions	Oscillator Strength	Visual rendering of the MOs
1	181β	d_{xy}	3.95	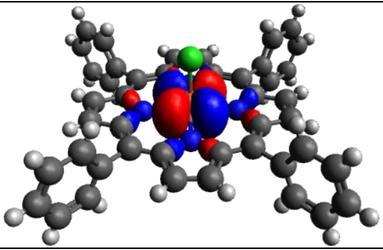
2	183β	d_{xz}	9.64	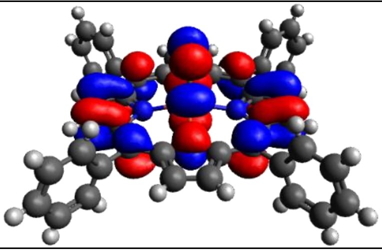
3	184β	d_{yz}	9.64	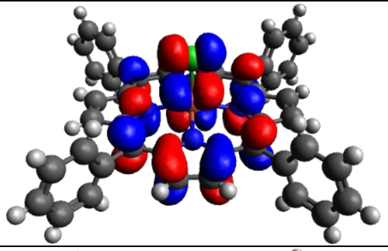
4	182β	d_{z2}	7.62	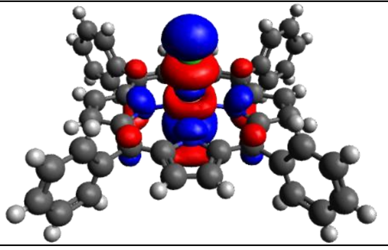

5	186β	d_{x2-y2}	40.64	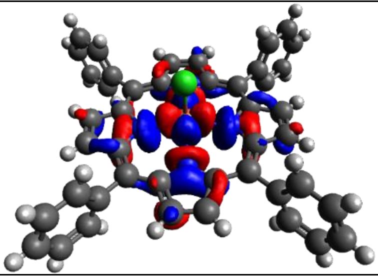
6	184α		2.21	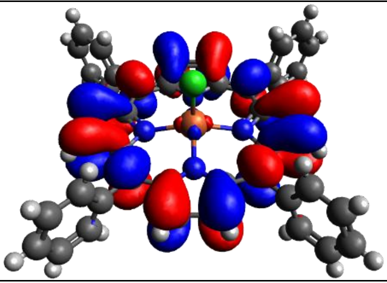

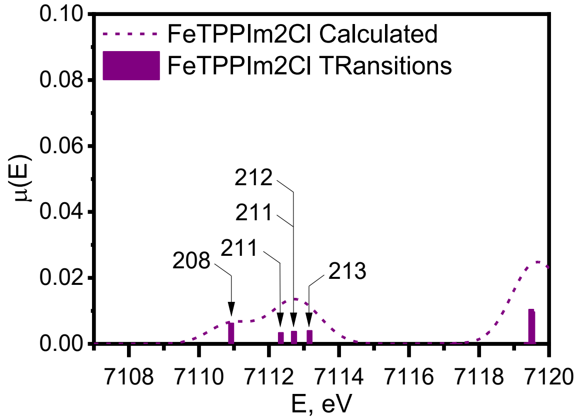

Figure S23. Calculated pre-edge feature and underlying transitions for $FeTPPIm_2Cl$

Table S17. Transitions, orbitals, oscillator strength for $FeTPPIm_2Cl$

Transition	Orbital	Major AO contributions	Oscillator Strength	Visual rendering of the MOs
1	208β	t2g	4.27	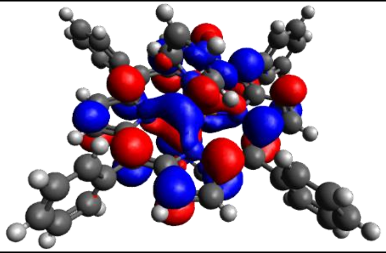
2	211α	d_{z2}	2.28	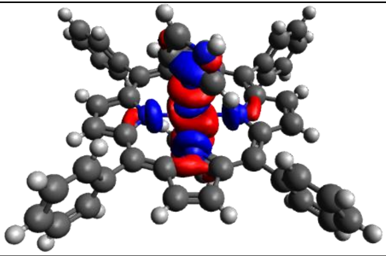
3	211β	d_{z2}	2.47	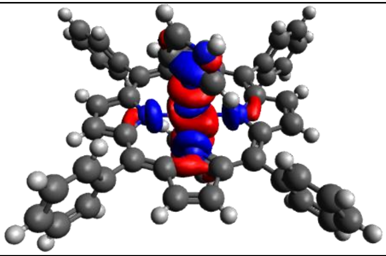
4	212α		2.53	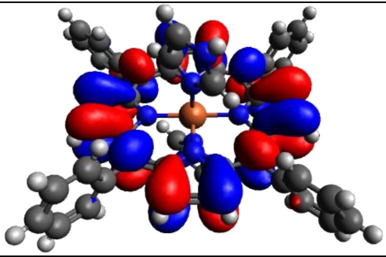

5	213β	d_{x2-y2}	2.67	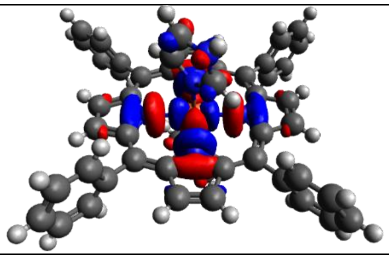
6	209α		0.02	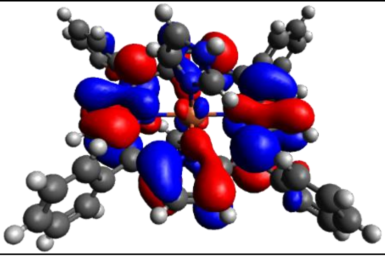

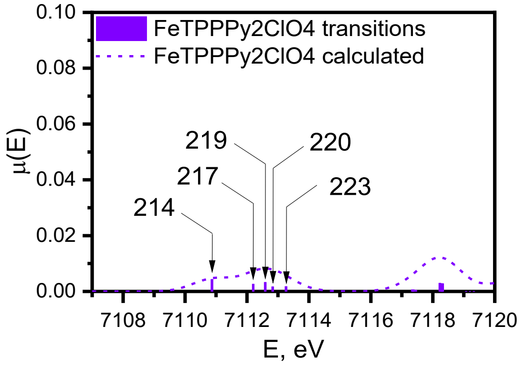

Figure S24. Calculated pre-edge feature and underlying transitions for $FeTPPPy_2ClO_4$

Table S18. Transitions, orbitals, oscillator strength for $FeTPPPy_2ClO_4$

Transition	Orbital	Major AO contributions	Oscillator Strength	Visual rendering of the MOs
1	214β	t2g	4.59	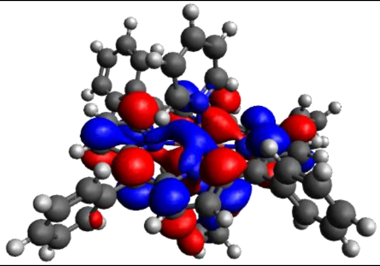
2	217α		2.47	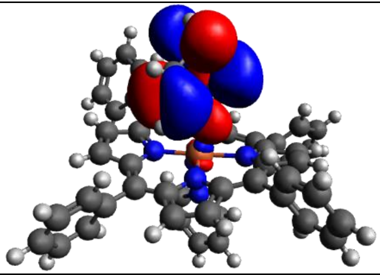
3	219β	d_{z2}	2.70	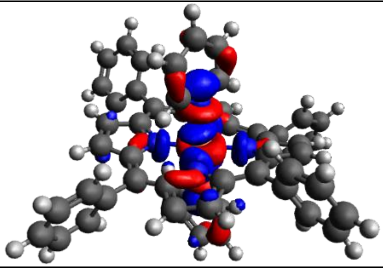
4	220α		2.71	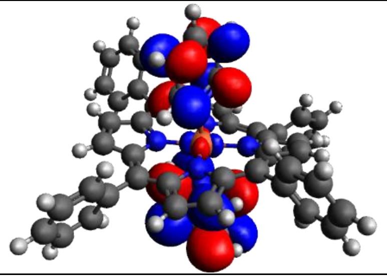

5	223β	d_{x2-y2}	2.87	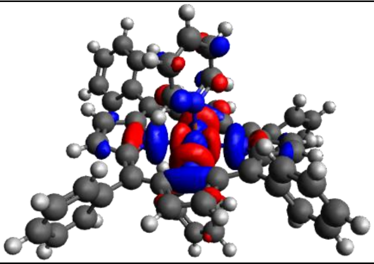

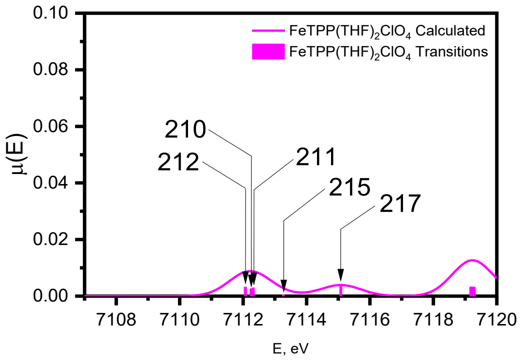

Figure S25. Calculated pre-edge feature and underlying transitions for $FeTPP(THF)_2ClO_4$

Table S19. Transitions, orbitals, oscillator strength for $FeTPP(THF)_2ClO_4$

Transition	Orbital	Major AO contributions	Oscillator Strength	Visual rendering of the MOs
1	212β	d_{xy}	4.59	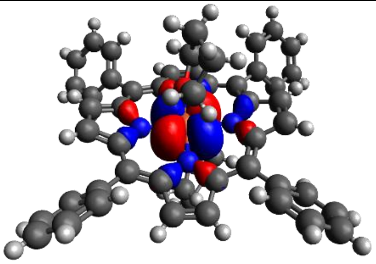
2	210β	d_{xz}	2.47	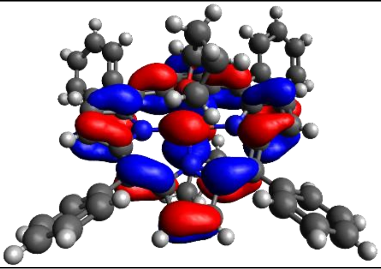
3	211β	d_{yz}	2.70	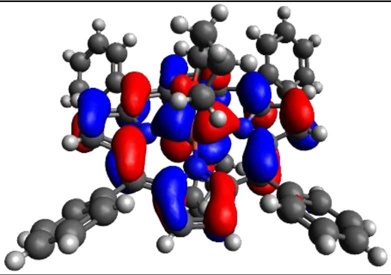
4	215β	d_{z2}	2.71	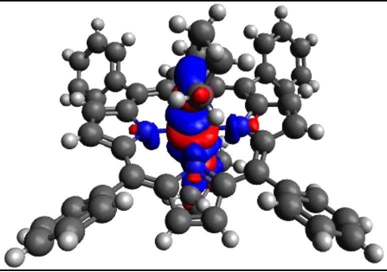

5	217β	d_{x2-y2}	2.87	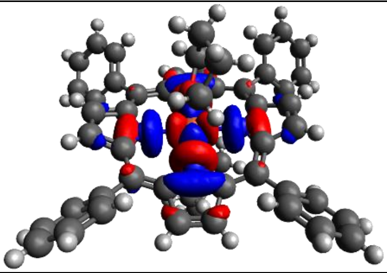

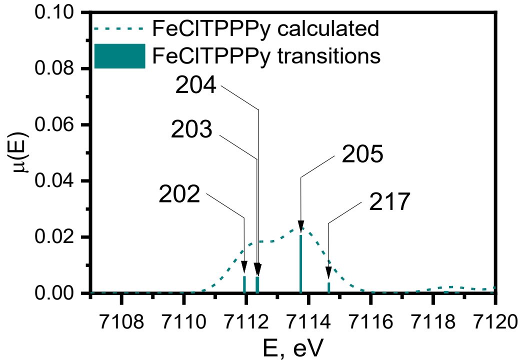

Figure S26. Calculated pre-edge feature and underlying transitions for FeClTPPPy

Table S20. Transitions, orbitals, oscillator strength for FeClTPPPy

Transition	Orbital	Major AO contributions	Oscillator Strength	Visual rendering of the MOs
1	202β	d_{xy}	4.25	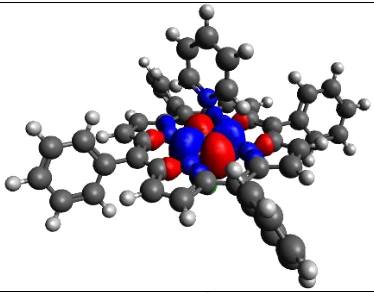
2	203β	d_{xz}	4.12	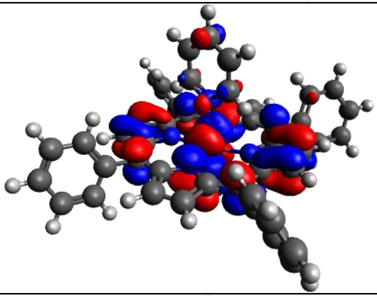
3	204β	d_{yz}	4.06	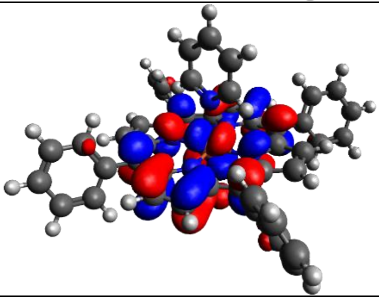

4	205β	d_{z2}	14.61	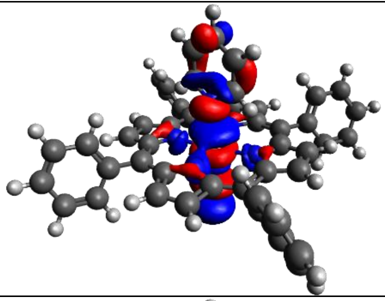
5	217β	d_{x2-y2}	2.66	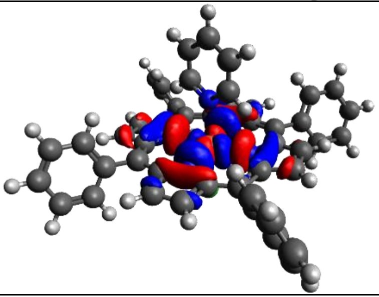

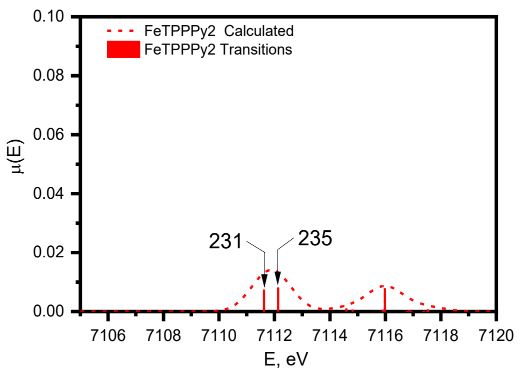

Figure S27. Calculated pre-edge feature and underlying transitions for $FeTPPPy_2$

Table S21. Transitions, orbitals, oscillator strength for $FeTPPPy_2$

Transition	Orbital	Major AO contributions	Oscillator Strength	Visual rendering of the MOs
2	231 α β	d_{z2}	5.15	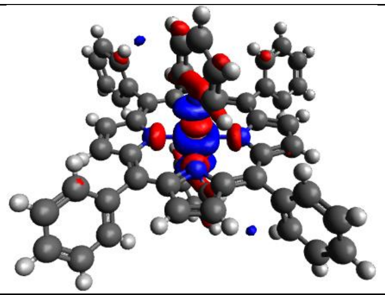
4	235 α β	d_{x2-y2}	5.65	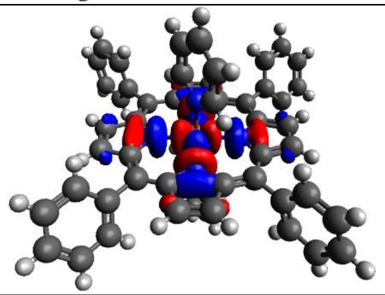

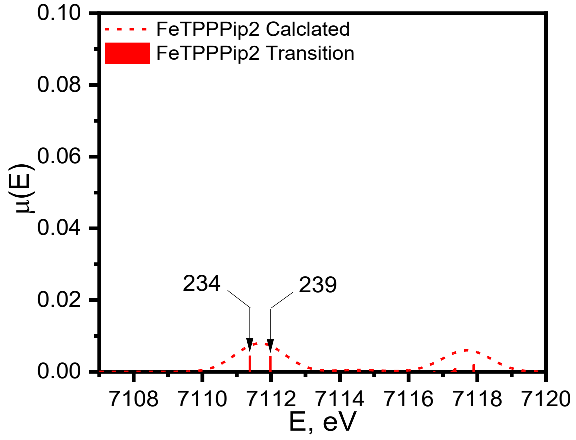

Figure S28. Calculated pre-edge feature and underlying transitions for $FeTPPPip_2$

Table S22. Transitions, orbitals, oscillator strength for $FeTPPPip_2$

Transition	Orbital	Major AO contributions	Oscillator Strength	Visual rendering of the MOs
2	234 α β	d_{z2}	5.65	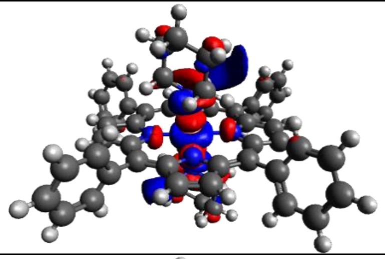
4	239 α β	d_{x2-y2}	5.56	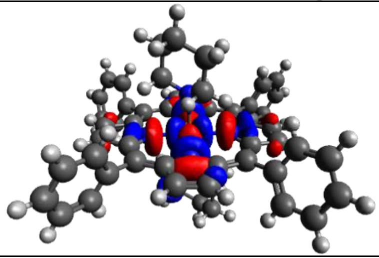

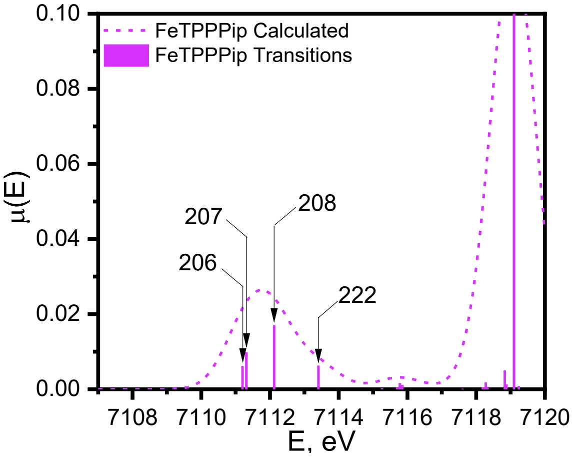

Figure S29. Calculated pre-edge feature and underlying transitions for FeTPPPip

Table S23. Transitions, orbitals, oscillator strength for FeTPPPip

Transition	Orbital	Major AO contributions	Oscillator Strength	Visual rendering of the MOs
1	206β	d_{xy}	4.31	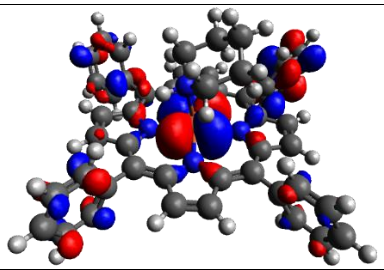
2	207β	d_{yz}	6.87	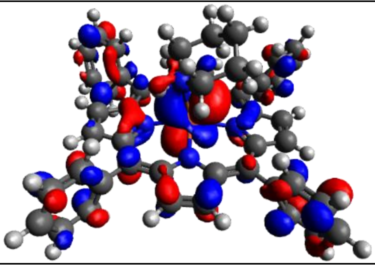
3	208β	d_{z2}	12.00	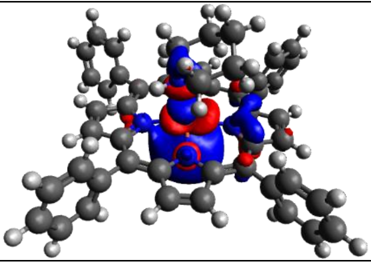

4	222β	d_{x2-y2}	4.37	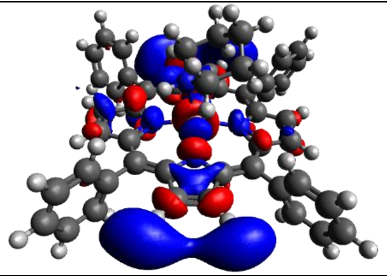

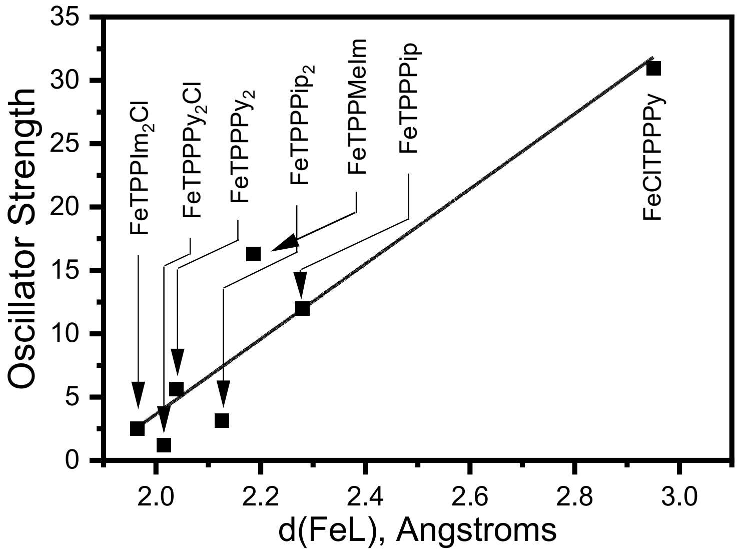

Figure S30. Dependence of oscillator strength for 1s → 3d$_{z2}$ transition from Fe-L bond length.

Synthesis: Reference complexes, FeClTPP, FeTPPIm$_2$Cl, FeTPPPy$_2$, FeTPPPip$_2$, MnClTPP, MnTPPIm$_2$Cl, MnTPPPip$_2$Cl, and MnClTPPPy were prepared according to the literature procedures.[3-8]

Characterization: A Cary-Varian UV-visible-NIR spectrophotometer equipped with a diffuse reflectance accessory (Harrick Scientific) was used to collect diffuse reflectance spectra. Samples were diluted with KBr and finely ground prior to measurement. Powder X-ray diffraction (PXRD) Patterns were recorded using Bruker D8 ADVANCE ECO Diffractometer.

X-ray Diffraction Studies:

Data for $C_{48}H_{28}ClMnN_4O_{16}Zr_3$ (**1**) and for $C_{56}H_{52}AgCl_2FeN_4O_{11}$ (**2**) were collected at 100 K on a Rigaku XtaLAB Synergy-S Dual Source diffractometer equipped with a PhotonJet Cu-microfocus source (λ = 1.54184 Å) and a HyPix-6000HE detector. Data reduction was performed with CrysAlisPro 65, and subsequent data processing was also performed in CrysAlisPro.[9] Using the SCALE3 ABSPACK scaling algorithm, empirical absorption

corrections were applied to the data for all two of the structures. Empirical and numerical (Gaussian) absorption corrections, determined by face indexing, and integration were applied to the data for (**1**) and (**2**). The structures were solved by applying the intrinsic phasing in SHELXT and refined by full-matrix least-squares techniques against F^2 (SHELXTL).[10-11]Anisotropic thermal factors were applied for all atoms except for the hydrogen atoms. H atoms were placed in idealized positions and refined using a riding model. For (**1**), unresolved disordered solvent molecules were removed using SQUEEZE in PLATON (4). The structural and refinement parameters can be found in Table 1; figures for (**1** & **2**) were generated using ORTEP. Numbers in parentheses are the errors in the least significant digits.

Table 1. X-Ray structural parameters for **(1) & (2)**

Experimental details	**(1)**	**(2)**
**Crystal data**		
Chemical formula	$C_{48}H_{28}ClMnN_4O_{16}Zr_3$	$C_{56}H_{52}AgCl_2FeN_4O_{11}$
M_r	1280.79	1191.63
Crystal system, space group	Hexagonal, *P6/mmm*	Triclinic, *P-1*
Temperature (K)	100(2)	100(2)
a, *b*, *c* (Å)	41.7982(5), 41.7982(5), 17.1794(3)	10.2562(1), 12.6509(2), 20.6626(3)
α, β, γ (°)	90., 90., 120.	105.141(1), 94.225(1), 104.340(1)
V (Å^3)	25992.8(8)	2479.68(6)
Z	6	2
Radiation type	CuKα	CuKα
μ (mm^{-1})	2.346	7.080
Crystal size (mm)	0.037 x 0.050 x 0.219	0.090 x 0.140 x 0.160
**Data collection**		
Diffractometer	Rigaku Synergy-S	Rigaku Synergy-S
Absorpt. corr.	numerical, Gaussian	numerical, Gaussian
T_{min}, T_{max}	0.564, 1.000	0.551, 1.000

No. of measured, independent and observed [$I > 2\sigma(I)$] reflections	156632, 7870, 6846	46990, 9992, 9265
Rint	0.109	0.033
(sin θ/λ)max (Å−1)	0.582	0.632
Refinement		
R[F > 2σ(F)], wR(F), S	0.084, 0.239, 1.04	0.035, 0.091, 1.08
No. of reflections	7870	9992
No. of params.	188	676
No. of restraints	60	0
H-atom treatment	constr	constr
Δρmax, Δρmin (eA^{-3})	1.805, -3.041	2.310, -0.999
CCDC number	2357076	2357077

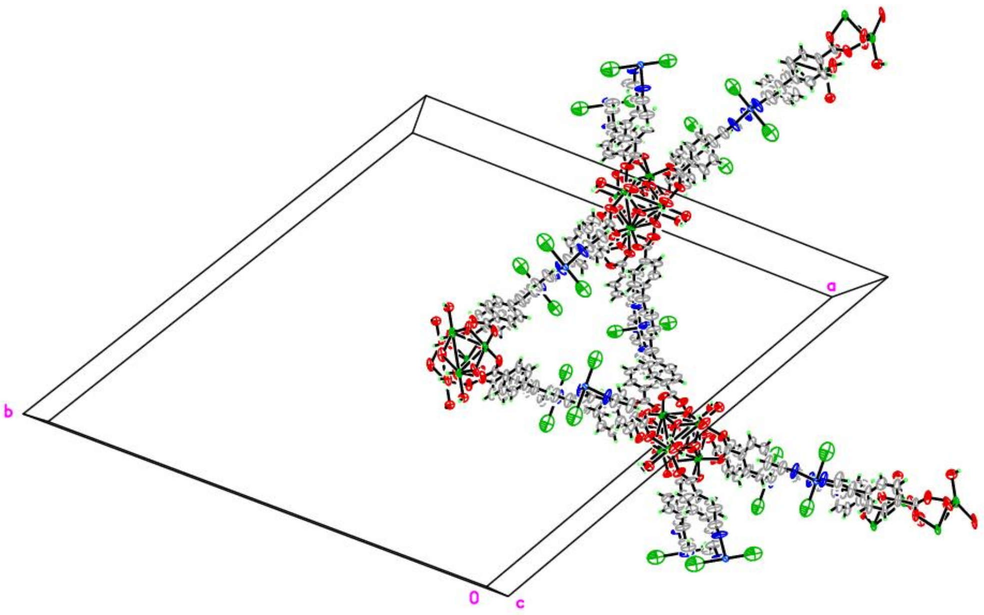

Figure S31. ORTEP partial packing diagram for (**1**), $C_{48}H_{28}ClMnN_4O_{16}Zr_3$. The ellipsoids were drawn at the 40% probability level.

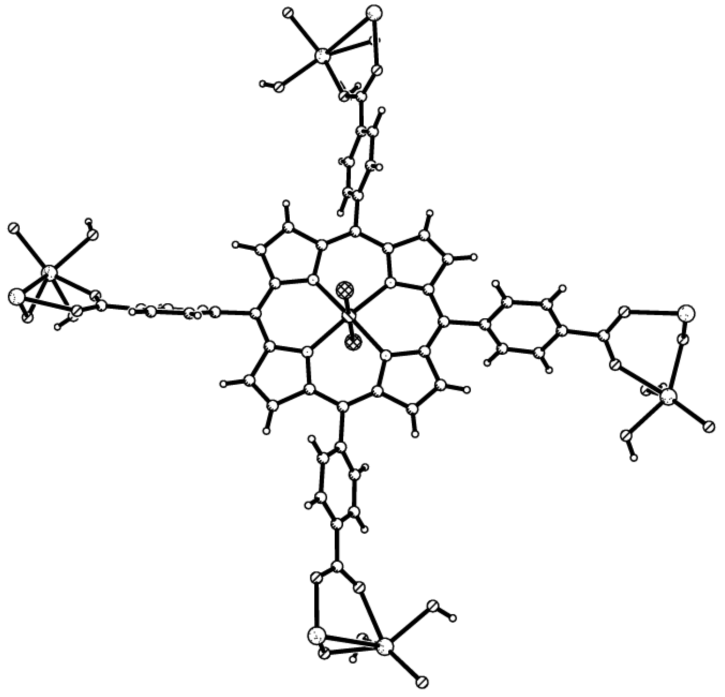

Figure S32. ORTEP diagram for (1), showing the planar macrocycle and the two Cl atoms trans to the ring system. **Cl_1 is there 72% of the time, Cl_2 is there 28% of the time.**

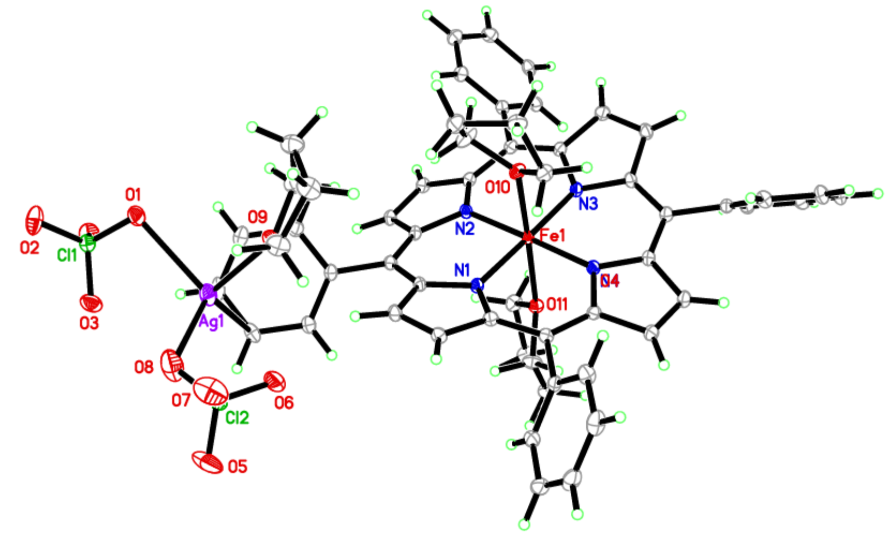

Figure S33. ORTEP plot for (**2**), $C_{56}H_{52}AgCl_2FeN_4O_{11}$. The ellipsoids were drawn at the 40% probability level.

References:

(1) Kucheryavy, Pavel; Lahanas, Nicole; Lockard, Jenny V.; Spectroscopic Evidence of Pore Geometry Effect on Axial Coordination of Guest Molecules in Metalloporphyrin-Based Metal Organic Frameworks. *Inorg. Chem.* **2018,** *57*, 3339–3347.
(2) Kucheryavy, Pavel; Lahanas, Nicole; Velasco, Ever; Sun, Cheng-Jun; Lockard, Jenny V.; Probing Framework-Restricted Metal Axial Ligation and Spin State Patterns in a Post-Synthetically Reduced Iron-Porphyrin-Based Metal–Organic Framework. *J. Phys. Chem. Lett.* **2016,** *7*, 1109–1115.
(3) Walker, F. Ann; Lo, Man-Wai; Ree, Molly T.; Electronic Effects in Transition Metal Porphyrins. The Reactions of Imidazoles and Pyridines with a Series of Para-Substituted Tetraphenylporphyrin Complexes of Chloroiron(III). *J. Am. Chem. Soc.* **1976,** *98*, 5552–5560.
(4) Radonovich, L. J.; Bloom, Allen; Hoard, J. L.; Stereochemistry of Low-Spin Iron Porphyrins. II. Bis(piperidine)-.alpha.,.beta.,.gamma.,.delta.-tetraphenylporphinatoiron(II). *J. Am. Chem. Soc.* **1972,** *94*, 2073–2078.

(5) Scheidt, W. Robert; Osvath, Sarah R.; Lee, Young Ja; Crystal and Molecular Structure of Bis(imidazole)(meso-tetraphenylporphinato)iron(III) chloride. A Classic Molecule Revisited. *J. Am. Chem. Soc.* **1987,** *109*, 1958–1963.
(6) Li, N.; Petrivcek, V.; Coppens, P.; Landrum, J.; Structure of Bis(pyridine)(5,10,15,20-tetraphenylporphyrinato)iron(II)--Pyridine Solvate, [Fe(C44H28N4)(C5H5N)2].2C5H5N. *Acta Crystallogr. C* **1985,** *41*, 902–905.
(7) Collman, James P.; Hoard, J. L.; Kim, Nancy; Lang, George; Reed, Christopher A.; Synthesis, Stereochemistry, and Structure-Related Properties of .alpha.,.beta.,.gamma.,.delta.-tetraphenylporphinatoiron(II). *J. Am. Chem. Soc.* **1975,** *97*, 2676–2681.
(8) Lahanas, Nicole; Kucheryavy, Pavel; Lalancette, Roger A.; Lockard, Jenny V.; Crystallographic identification of a series of manganese porphyrin complexes with nitrogenous bases. *Acta Crystallogr. C* **2019,** *75*, 304–312.
(9) RigakuOD, (2020), CrysAlis PRO. Rigaku Oxford Diffraction Ltd, Yarnton, Oxfordshire, England.
(10) Sheldrick, George; SHELXT - Integrated space-group and crystal-structure determination. *Acta Crystallographica Section A* **2015,** *71*, 3–8.
(11) Sheldrick, George; Crystal structure refinement with SHELXL. *Acta Crystallogr. C* **2015,** *71*, 3–8.
